# Monitoring Alien Species Diversity in Ballast Water Based on Environmental DNA Metabarcoding

**DOI:** 10.1002/ece3.72320

**Published:** 2025-10-14

**Authors:** Hanglei Li, Hui Jia, Jingbo Peng, Xiaofeng Peng, Zhipeng Ren, Hui Zhang

**Affiliations:** ^1^ Laboratory of Marine Ecology and Environmental Sciences, Institute of Oceanology Chinese Academy of Sciences Qingdao China; ^2^ Laboratory for Marine Ecology and Environmental Science Qingdao Marine Science and Technology Center Qingdao China; ^3^ University of Chinese Academy of Sciences Beijing China; ^4^ Dongjiakou Maritime Safety Administration Qingdao China; ^5^ Shandong Maritime Safety Administration Qingdao China

**Keywords:** alien species monitoring, ballast water biodiversity, environmental DNA metabarcoding, marine biosecurity

## Abstract

Invasive alien species pose serious threats to ecosystems, public health, and socio‐economic systems, with ballast water discharge serving as a major pathway for their introduction. Organisms that survive the harsh conditions inside ballast tanks may establish populations in recipient ports, where they can disrupt native biodiversity. Therefore, effective monitoring of ballast water is essential for reducing the risk of biological invasions. This study applied environmental DNA (eDNA) metabarcoding to investigate the biological communities in ballast water from ships arriving at Dongjiakou Port, Qingdao, with a particular focus on alien species. Three universal primer sets targeting the 18S V4, 18S V9, and 12S regions were used to amplify and sequence DNA from phytoplankton, invertebrates, and fish. In total, nine ballast water samples were collected from six ships originating from the South China Sea, Seto Inland Sea, Taiwan Strait, and the Yellow Sea. Bioinformatic analysis revealed 16 alien phytoplankton species, 13 alien invertebrate species, and 12 alien fish species, including three invasive species: *Styela clava* , *Lates calcarifer* , and *Anguilla anguilla* . Species composition varied considerably among tanks on the same ship, whereas ballast water location and age had no significant effect on composition across different ships. These results demonstrate the potential of eDNA metabarcoding as an efficient, noninvasive approach for monitoring ballast water biodiversity and alien species. Such insights are valuable for informing policy and management strategies to curb the spread of invasive species through shipping networks.

## Graphical abstract

This study applied environmental DNA (eDNA) metabarcoding to assess alien species in ballast water from ships at Dongjiakou Port, Qingdao, identifying 41 alien species including three invasives. The findings demonstrate the effectiveness of eDNA as a noninvasive tool for monitoring ballast water biodiversity and offer valuable insights for managing the spread of invasive species through shipping.

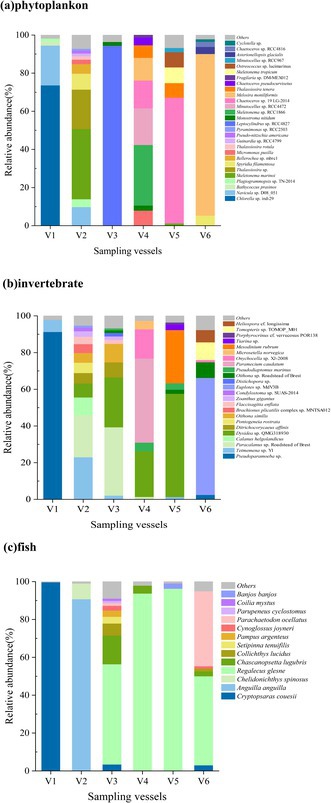

## 1 Introduction

Alien species are taxa that have been deliberately or unintentionally introduced beyond their native biogeographic ranges through anthropogenic activities (Blackburn et al. 2014). When such species are able to tolerate novel environmental stressors, establish self‐sustaining populations, and subsequently enter the dispersal phase, they are often reclassified as invasive species, which are widely recognized as posing substantial risks to native biodiversity, ecosystem functioning, and associated services (Blackburn et al. 2014; Lei 2008). At this stage, management becomes considerably more complex, and the ecological and socio‐economic damage they inflict is frequently irreversible. Consequently, early monitoring, rapid response, and preventive interventions constitute the effective strategies for mitigating biological invasions (Lei 2008; Vander Zanden et al. 2010). Such proactive measures not only constrain the spatial spread of alien species but also reduce the magnitude of their adverse ecological and socio‐economic impacts (Vander Zanden et al. 2010). It is noteworthy that the initial introduction phase, during which alien species are transported through multiple pathways, represents a critical window of opportunity for management. Targeted and timely monitoring during this stage is particularly important, as it can effectively prevent alien populations from establishing and transitioning into invasive species, thereby averting long‐term and potentially irreversible ecological disruption and socio‐economic costs.

Alien marine species are introduced into novel environments primarily through human‐mediated activities, including commercial shipping, aquaculture, the seafood trade, the aquarium industry, and intentional or accidental anthropogenic releases (Kerr et al. 2005; Martin and Coetzee 2011). Among these pathways, ballast water discharge associated with maritime transport is widely recognized as one of the most significant vectors facilitating the introduction of non‐native marine taxa (Cope et al. 2015). Ballast water, which is indispensable for maintaining vessel stability, contains diverse assemblages of planktonic and benthic organisms, some of which are capable of withstanding the extreme physicochemical conditions within ballast tanks (Ardura, Rick, et al. 2021; García‐Garay et al. 2018; Marangoni et al. 2001). Upon discharge into coastal environments, these stress‐tolerant species exhibit an increased likelihood of establishment in port ecosystems, thereby elevating the probability of marine biological invasions (Ardura, Martinez, et al. 2021). Given these risks, the development and application of advanced monitoring technologies are imperative for the timely detection and accurate assessment of alien biodiversity within ballast water.

Conventional morphological identification methods are often labor‐intensive, time‐consuming, and subject to considerable error, particularly when distinguishing morphologically similar taxa (Briski et al. 2012; DiBacco et al. 2012; Simard et al. 2011). In contrast, molecular approaches, such as environmental DNA (eDNA) metabarcoding, provide a more efficient and accurate alternative. This technique extracts and sequences DNA directly from aquatic environments, thereby overcoming many of the limitations associated with traditional approaches (Pukk et al. 2021; Zhang and Xian 2020). eDNA metabarcoding has been widely employed in invasion biology, with applications ranging from the early monitoring of invasive populations (Clusa et al. 2021; Jeunen et al. 2022) to the reconstruction of invasion pathways (Mahon et al. 2014; Rey et al. 2019). More recently, its utility in assessing biodiversity within ballast water has received increasing attention. For example, one study demonstrated the validity of eDNA metabarcoding by directly comparing its outcomes with those derived from conventional visual surveys, thereby confirming its reliability for detecting non‐indigenous species (Zaiko et al. 2015). Similarly, analysis of ballast water from eleven ships successfully identified the alien copepod *Oithona davisae* , underscoring the role of ballast water as a critical vector for species introductions (Rey et al. 2019). Another investigation applied eDNA metabarcoding to characterize microbial assemblages in ballast tanks, revealing a decline in overall biodiversity with increasing voyage duration, while resilient and potentially harmful taxa persisted (Ardura, Martinez, et al. 2021). Collectively, these studies highlight not only the versatility and effectiveness of eDNA metabarcoding in monitoring alien taxa in ballast water but also its promise as a tool for developing ecological indicators to inform management strategies aimed at mitigating marine biological invasions.

However, the current application of eDNA metabarcoding in ballast water studies remains constrained in both taxonomic scope and trophic breadth. To date, most investigations have primarily targeted microorganisms and lower trophic groups, including bacteria (Gerhard and Gunsch 2019; Lymperopoulou and Dobbs 2017), viruses (Kim et al. 2015), phytoplankton (Queiroz et al. 2024), zooplankton (McCollin et al. 2008), and invertebrates (Chan et al. 2015), whereas larger organisms, such as fish, have received comparatively limited attention (Wonham et al. 2000). The present study extends previous research in three principal ways. First, it simultaneously integrates multiple taxonomic groups across distinct trophic levels—namely phytoplankton (primary producers), invertebrates (primary consumers), and fish (higher‐order consumers)—thereby providing a more holistic characterization of ballast water biodiversity. Second, it places emphasis on taxa with well‐documented ecological traits and survival strategies, such as the formation of dormant stages in phytoplankton and invertebrates (Ellegaard and Ribeiro 2018; Radzikowski 2013), which substantially enhance their likelihood of successful transport, persistence, and eventual colonization. Third, it explicitly focuses on fish eggs as cryptic yet ecologically significant propagules. The presence of fish eggs in ballast tanks may facilitate the survival and dispersal of fish species, thus posing substantial invasion risks (Kornis et al. 2012). These three taxonomic groups are ecologically interconnected and constitute essential energy pathways within marine food webs. Monitoring them concurrently allows for a more comprehensive assessment of the potential for alien species establishment via ballast water discharge. This integrative approach not only broadens the taxonomic and trophic coverage of eDNA‐based monitoring but also provides novel insights for strengthening ballast water biosecurity and informing invasive species management strategies.

Dongjiakou Port (35°39′–35°33′ N, 119°41′–119°48′ E, Qingdao City, Shandong Province, China) constitutes a critical component of Qingdao Port, one of China's national hub ports. As the location of the country's largest 400,000‐ton iron ore terminal, it accommodates the routine berthing of vessels of this capacity, thereby reinforcing its strategic importance in international maritime trade (Yang 2017; Yu et al. 2017). The port plays a pivotal role in regional economic development, functioning as a major center for industrial exchange and logistics and serving as a driving force behind sustained economic growth. Between 2018 and 2021, the number of vessels calling at Dongjiakou Port increased steadily, reflecting its expanding influence within the regional economy (Liu et al. 2023). However, this intensification of shipping activity has concomitantly elevated the risk of alien species introductions via ballast water discharge. As the frequency and volume of ballast water release rise, so too does the probability of transporting non‐indigenous organisms, with potentially severe consequences for marine biodiversity and local fisheries. It is therefore noteworthy that effective monitoring and management strategies are urgently required to mitigate the ecological risks associated with ballast water discharge. Strengthening biosecurity measures at Dongjiakou Port will be essential not only for safeguarding regional marine ecosystems but also for supporting the sustainable development of coastal socio‐economic systems.

In this study, eDNA metabarcoding was employed to analyze ballast water samples from both domestic and international vessels arriving at Dongjiakou Port. The primary objectives were threefold: (1) to characterize the species composition and biodiversity of phytoplankton, invertebrates, and fish within ballast water; (2) to evaluate the influence of ballast water source location, water age, and intra‐vessel tank variation on species assemblages; and (3) to monitor alien taxa while exploring management strategies aimed at mitigating their potential ecological impacts. We hypothesized that ballast water location, water age, and intra‐vessel tank variation would exert significant effects on community composition. By concurrently examining multiple trophic levels in relation to these environmental variables, this study provides a comprehensive assessment of ballast water biodiversity and underscores its critical role as a vector for the introduction and establishment of alien marine species.

## 2 Materials and Methods

### 2.1 Sample Collection

**FIGURE 1:**
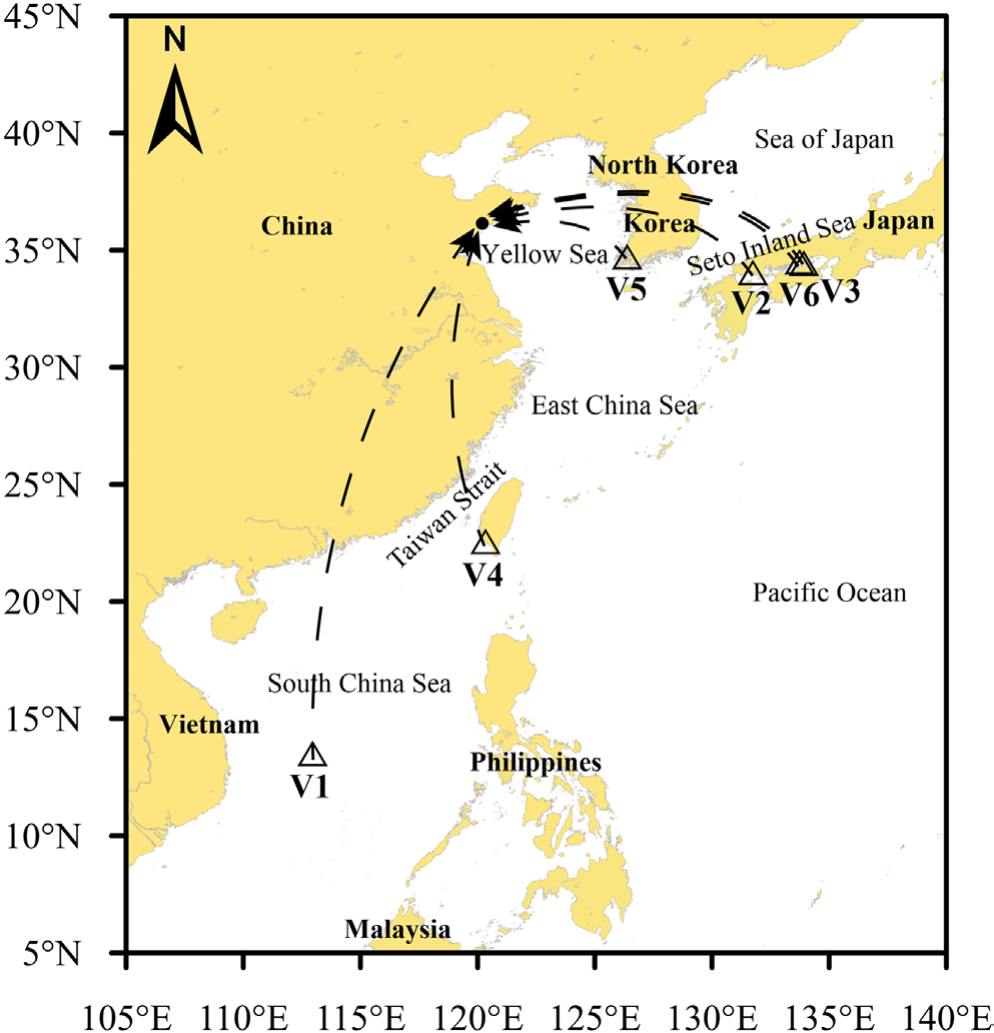
Locations of ballast water uptake in the ships.

Between February and June 2024, ballast water samples were collected from six vessels berthed at Dongjiakou Port. Sterilized 1000 mL brown plastic bottles were employed for sampling, with each bottle containing 100 mL of a commercial storage solution (Tiandz Inc., Beijing, China) to preserve sample integrity (Kumar et al. 2020). For each vessel, three replicate samples were collected, and three blank controls consisting of ultra‐pure water were prepared to ensure data reliability. To examine potential intra‐vessel variability in biological composition, more detailed sampling was undertaken for two ships (V5 and V6), with samples collected from multiple ballast tanks. All procedures strictly followed the International Maritime Organization (IMO) Guideline (G2) (IMO International Maritime Organization 2004). Immediately after collection, samples were sealed and stored in darkness until laboratory filtration. The geographic locations where ballast water was loaded for each vessel are presented in Figure 1. Notably, ballast water for V1 was loaded in the central South China Sea, whereas vessels V3 and V6 loaded ballast water at nearly identical sites, resulting in overlapping points on the map. Detailed metadata for each vessel and sampling protocol are summarized in Table 1.

**TABLE 1:** Detailed information about the ships.

Station	IMO number	Ship name	Date of uptake	Sampling date	Ballast tank number and sampling volume	Location of uptake	Sea area	Ballast water age
V1	9735945	Orion	2.21	3.11	BWT (3 L)	13.516° N, 112.955° E	Central South China Sea	19 days
V2	9485904	Safeen al amal	4.4	4.18	BWT (3 L)	34.050° N, 131.750° E	Seto Inland Sea	14 days
V3	9615042	Friendly islands	5.17	5.24	DBT (3 L)	34.450° N, 133.927° E	Seto Inland Sea	7 days
V4	9146558	Sea alice	5.10	6.3	BWT (3 L)	22.562° N, 120.330° E	Taiwan Strait	24 days
V5	8747654	Jin yang guan	5.30	6.3	FPT (2 L), APT (1 L)	34.734° N, 126.379° E	South Yellow Sea	4 days
V6	9533335	Mandarin river	5.28	6.5	FPT (1 L), APT (1 L), DBT (1 L)	34.514° N, 133.737° E	Seto Inland Sea	8 days

*Note:* “Sea area” refers to the location where ballast water was taken on, and “Ballast water age” indicates the duration (in days) the ballast water remained in the tank from intake to sampling, prior to discharge at Dongjiakou Port.

Abbreviations: APT, Aft Peak Tank; BWT, Ballast Water Tank; DBT, Double Bottom Tank; FPT, Fore Peak Tank.

Among all vessels arriving at Dongjiakou Port during the study period, several carried no ballast water and were therefore excluded from sampling. The six vessels selected for analysis were the first to arrive with ballast water onboard. Due to variability in arrival times, sampling was conducted opportunistically from all ships carrying ballast water, without restrictions on their specific sailing routes. It should be acknowledged that ballast water loading at a single site—such as the South China Sea, the Yellow Sea and the Taiwan Strait—cannot be considered fully representative of the entire region. Rather, these loading sites are treated as indicative of general ballast water uptake zones, providing useful but not exhaustive insights into source‐area biodiversity.

### 2.2 DNA Extraction and Filtration

Filtration was performed under controlled laboratory conditions using Sterivex‐GP filters (pore size: 0.22 μm; EMD Millipore Corp., Billerica, MA, USA) in combination with a peristaltic pump (Masterflex L/S, ThermoFisher Scientific, Waltham, MA, USA) operating at 60 rpm. Following filtration, DNA preservation solution (Tiandz, Beijing, China) was added directly to each filter column to stabilize nucleic acids. Filters were subsequently stored at −20°C until environmental DNA (eDNA) extraction. These procedures were implemented to ensure sample integrity and minimize DNA degradation prior to molecular analyses.

Total DNA was extracted from all filter membranes using the DNeasy PowerSoil Pro Kit (Qiagen, Germany) following the manufacturer's protocol. DNA concentration was quantified with a NanoDrop spectrophotometer (Thermo Fisher Scientific, USA), and quality was assessed via 0.8% agarose gel electrophoresis.

To generate representative samples for each vessel, ballast water collected from a single ballast tank (i.e., vessels V1, V2, V3, and V4) was pooled following filtration but prior to DNA extraction. This pooling strategy was designed to capture the overall species composition within an individual ballast tank while maintaining methodological efficiency. In contrast, samples obtained from multiple ballast tanks on the same vessel (i.e., V5 and V6) were processed independently to evaluate the influence of intra‐vessel tank‐specific environmental conditions on community composition. In total, nine ship‐derived samples and one blank control (generated by pooling the three blank replicates after filtration) were retained for downstream molecular analyses, resulting in ten final samples. Detailed information on ballast tank sampling conditions and sample volumes is provided in Table 1. To ensure data integrity and minimize the risk of contamination, all equipment and instruments were thoroughly sterilized prior to use in accordance with rigorous quality‐control protocols.

### 2.3 PCR Amplification, Sequencing, and Sequence Analysis

Three universal primer sets were employed in this study (Table 2): primers targeting the V4 and V9 regions of the eukaryotic ribosomal large subunit (Stoeck et al. 2010; Xue et al. 2018) and primers targeting the mitochondrial 12S rRNA gene (Miya et al. 2015). These primers were selected for their ability to amplify DNA from phytoplankton, invertebrates, and fish, respectively, thereby facilitating a comprehensive characterization of ballast water biodiversity. Library preparation was conducted using a two‐step PCR protocol (Bohmann et al. 2022). For each sample, triplicate PCR reactions were performed and subsequently pooled in equal volumes to minimize stochastic amplification bias. Each reaction had a final volume of 25 μL, consisting of 5 μL of 5× reaction buffer, 5 μL of 5× High GC buffer, 2 μL of dNTPs (10 mM), 1 μL of forward primer (10 μM), 1 μL of reverse primer (10 μM), 2 μL of DNA template, 8.75 μL of ddH2O, and 0.25 μL of Q5 high‐fidelity DNA polymerase. Thermal cycling conditions were optimized according to target taxa, beginning with an initial denaturation at 98°C for 5 min, followed by 30 cycles of denaturation at 98°C for 30 s, annealing at 46°C (phytoplankton), 50°C (invertebrates), or 58°C (fish) for 30 s, and extension at 72°C for 45 s, with a final extension at 72°C for 5 min. Negative controls were included in every PCR batch to monitor for potential contamination. Samples exhibiting amplification in negative controls were excluded from subsequent analyses to ensure data reliability.

**TABLE 2:** Primers for three gene markers.

PCR assay	Target taxa	Primer name	Oligonucleotide sequence	Target length	Primer reference
18Sv4	Phytoplankton	547F	CCAGCASCYGCGGTAATTCC	420 bp	Stoeck et al. (2010)
		V4R	ACTTTCGTTCTTGATYRA		
18Sv9	Invertebrate	1380F	CCCTGCCHTTTGTACACAC	131 bp	Xue et al. (2018)
		1510R	CCTTCYGCAGGTTCACCTAC		
12S	Fish	MiFish‐U	ACACTCTTTCCCTACACGACGCTCTTCCGATCTNNNNNNGTCGGTAAAACTCGTGCCAGC	160–180 bp	Miya et al. (2015)
			GTGACTGGAGTTCAGACGTGTGCTCTTCCGATCTNNNNNNCATAGTGGGGTATCTAATCCCAGTTTG		

Fluorescence quantification of PCR products was conducted using a Microplate Reader (BioTek FLx800) and the Quant‐iT PicoGreen dsDNA Assay Kit. Based on quantification results, amplicons were proportionally pooled according to the sequencing depth required, rather than normalized to a fixed concentration, thereby ensuring a balanced distribution of sequencing reads across samples. Sequencing libraries were prepared using the KAPA DNA HyperPlus Library Preparation Kit (KAPA, KK8514) and subsequently subjected to paired‐end sequencing on the Illumina NovaSeq platform. A PhiX control was spiked in at a ratio of 30% to increase base diversity during sequencing runs. All raw sequence data were generated in FASTQ format and retained for downstream analyses. For 18S rRNA gene amplicons, bioinformatic processing was performed using the DADA2 package, which resolves high‐resolution amplicon sequence variants (ASVs) while minimizing sequencing error rates (Callahan et al. 2016). For 12S rRNA gene amplicons, VSEARCH was employed to cluster reads into operational taxonomic units (OTUs) based on sequence similarity thresholds, thereby enabling robust taxonomic assignment (Rognes et al. 2016).

For DADA2‐based analysis, primer sequences were first trimmed using the qiime cutadapt trim‐paired command, and non‐matching sequences were filtered out. Quality control, denoising, sequence merging, and chimera removal were subsequently conducted using the qiime dada2 denoise‐paired command. The resulting sequences were clustered at 100% similarity to generate ASVs and corresponding abundance tables. After denoising, ASV feature sequences (representing unique biological sequences) and their abundance data were merged. To enhance data reliability and reduce sequencing noise, ASVs represented by a single read were excluded from downstream analyses.

For VSEARCH‐based analysis, primer sequences were trimmed using cutadapt. Paired‐end reads were merged with the fastq_mergepairs module, and quality control was performed with the fastq_filter module. Duplicate sequences were removed using the derep_fulllength module. Clustering was performed in two steps: first, sequences were clustered at 98% similarity using the cluster_size module to group highly similar reads, followed by chimera removal with the uchime_denovo module. High‐quality sequences were then clustered at 97% similarity with the cluster_size module to generate representative sequences and OTU tables. As with ASVs, singletons (OTUs represented by only one sequence) were excluded to minimize spurious diversity estimates (Lymperopoulou and Dobbs 2017). To further ensure data integrity, ASVs/OTUs detected in negative controls were treated following the contamination‐filtering protocol described by a previous study (Pagenkopp Lohan et al. 2016), thereby reducing the likelihood of incorporating contamination artifacts into the final dataset.

### 2.4 Data Analysis

#### 2.4.1 Taxonomic Annotations and Species Composition Statistics

Taxonomic assignment of 18S rRNA V4 and V9 sequence data was conducted using the NCBI nucleotide database, whereas 12S rRNA sequence data were annotated against the MitoFish database. To enhance the accuracy of species identifications, all annotations were further cross‐validated against the World Register of Marine Species (WoRMS, http://www.marinespecies.org/) and AlgaeBase (http://www.algaebase.org/; Ardura, Martinez, et al. 2021). These supplementary databases were selected for their comprehensive coverage of marine phytoplankton, invertebrates, and fish, thereby ensuring that taxonomic assignments were both robust and consistent with the ecological focus of the present study.

Because the present study focused specifically on marine phytoplankton, invertebrates, and fish, non‐target taxa—including freshwater and terrestrial organisms, marine fungi, viruses, and categories annotated as “unclassified,” “uncultured,” “uncultivated,” “unknown,” or “metagenomic”—were excluded from further analyses. The remaining sequences were merged such that multiple sequences corresponding to the same species were consolidated into a single ASV/OTU, thereby reducing redundancy and ensuring that each unit represented a unique species. To validate species distributions, taxonomic records were cross‐checked against multiple authoritative databases, including FishBase (https://www.fishbase.se/search.php), SeaLifeBase (http://www.sealifebase.org/), the *Checklist of Marine Biota of China Seas* (Liu 2008), the Global Biodiversity Information Facility (https://www.gbif.org/), the Taiwan Fish Database (https://fishdb.sinica.edu.tw/), and the China Species Library (https://species.sciencereading.cn/biology/v/biologicalIndex/122HTML). Species not reported from the ballast water uptake regions were removed to maintain spatial relevance and improve the ecological validity of the dataset.

Taxonomic profiles were generated for the three focal groups—phytoplankton, invertebrates, and fish—at the phylum, class, and genus levels. For each vessel, species composition was summarized, with particular emphasis on taxa exhibiting relative abundances greater than 1%, thereby highlighting ecologically significant contributors. Distribution patterns and relative abundance differences across samples were visualized using histograms, providing a detailed overview of taxonomic diversity and ecological trends within the dataset.

#### 2.4.2 Diversity Analysis

To evaluate community diversity and structure, rarefaction curves were constructed to assess whether sequencing depth was sufficient to capture within‐sample diversity. Three α‐diversity indices were calculated for each ship and ballast tank: the Chao1 index (species richness); the Shannon index (overall diversity); and the Pielou index (species evenness). Heatmaps were generated to visualize community structures and highlight differences in species abundance across ships and ballast tanks. UpSet plots were constructed to quantify the total number of species, shared species (across ships and ballast tanks), and unique species, thereby elucidating patterns of overlap and distinctiveness in species composition. The effects of ballast age and ballast location on species diversity were assessed using Permutational Multivariate Analysis of Variance (PERMANOVA), a robust statistical method for testing differences in multivariate data. In addition, non‐metric multidimensional scaling (NMDS) was applied to explore differences in species assemblages among ballast tanks, providing an ordination‐based visualization of ecological dissimilarity across samples.

Bar plots were generated using Origin 2021, while rarefaction curves, heatmaps, UpSet plots, and NMDS plots were produced in R (version 4.0.3) utilizing the ggplot2, pheatmap, and UpSetR packages. These visualization approaches provided clear and systematic representations of species diversity, taxonomic composition, and distributional differences across samples, thereby facilitating comparative analyses of ecological patterns.

#### 2.4.3 Alien Species Statistics

This analysis aimed to evaluate the potential invasion risks associated with ballast water discharge from vessels operating in the Qingdao area. The assessment focused on monitoring non‐indigenous taxa among phytoplankton, invertebrates, and fish, with particular attention to their distributional patterns and invasion potential. The invasive status of these species in China was confirmed using *China's Invasive Alien Species* (Xu and Qiang 2018). For each identified alien taxon, relative abundance and source‐region information were analyzed, and an extensive review of the scientific literature was conducted to determine records of prior occurrence in Qingdao as well as potential ecological and economic consequences.

By integrating invasive status, spatial distribution data, and literature‐based evaluations, this study establishes a scientific foundation for the risk assessment of alien species introductions via ballast water. Furthermore, these findings provide valuable insights for the development of targeted management strategies aimed at reducing the likelihood of future invasions in the Qingdao region.

## 3 Results

### 3.1 Sequencing Results and Species Composition

High‐throughput sequencing of environmental DNA (eDNA) from the 10 collected samples yielded substantial read counts across the three primer sets. Specifically, the 18S V4 primer produced between 76,930 and 131,293 reads per sample, with a mean of 102,068.4 and a cumulative total of 1,020,684 reads. The 18S V9 primer generated between 72,413 and 136,709 reads, with an average of 111,926.8 and a total of 1,119,268 reads. The 12S primer yielded between 51,342 and 120,873 reads, with a mean of 78,266.8 and a cumulative total of 782,668 reads (Table S1). Following quality control and taxonomic refinement, non‐target ASVs and OTUs—including those derived from negative controls, as well as terrestrial, freshwater, fungal, viral, vertebrate, and other irrelevant taxa—were removed. Furthermore, redundant sequences representing the same taxon were merged to ensure accurate estimates of species diversity. After these filtering steps, 76 phytoplankton, 70 invertebrate, and 100 fish ASVs/OTUs were retained. Detailed taxonomic information for the three groups is summarized in Table S2, providing insights into the diversity of species identified from the eDNA samples.

**FIGURE 2:**
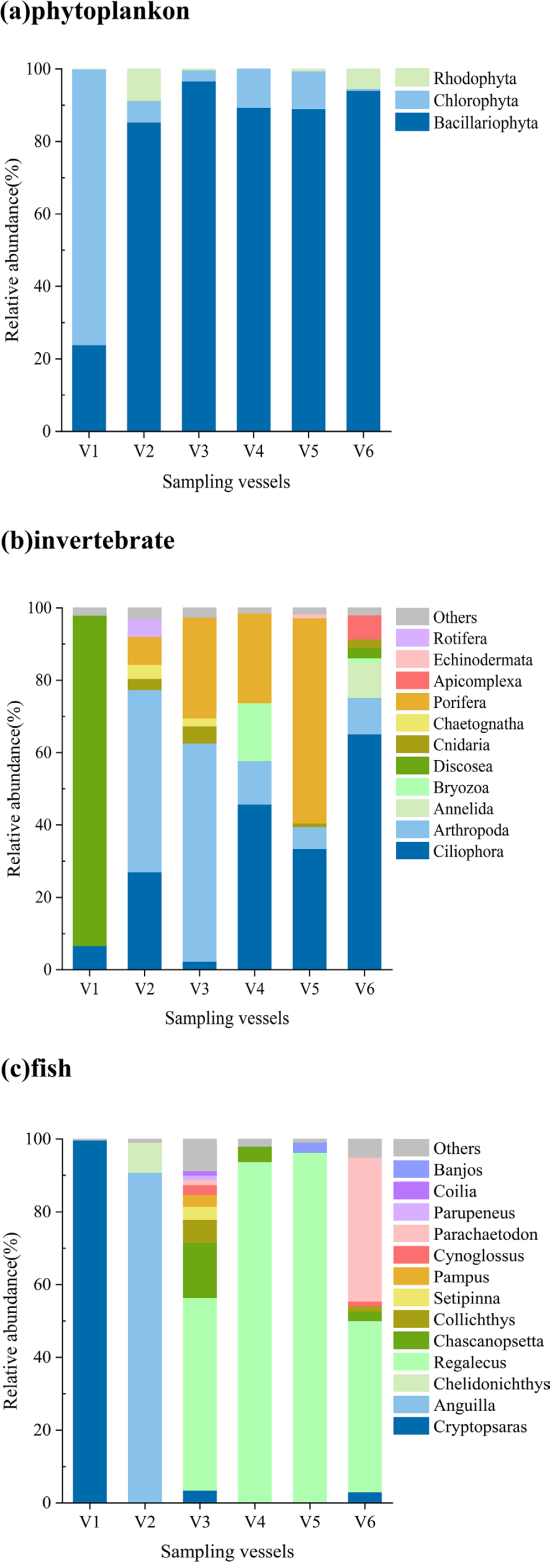
Relative abundance of groups in ballast water sample at the phylum, phylum, and genus levels.

**FIGURE 3:**
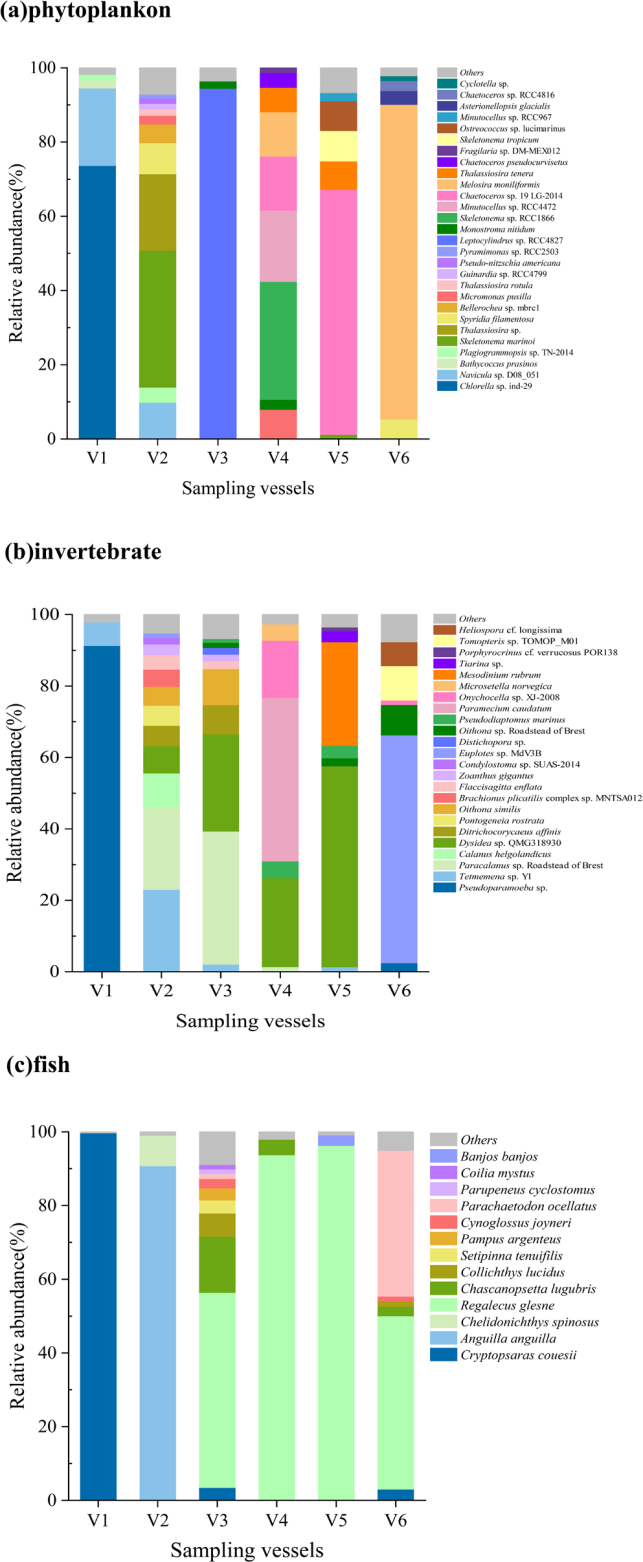
Relative abundance of groups in ballast water samples at the species levels.

Based on taxonomic information verified through WoRMS and AlgaeBase, the 76 phytoplankton species identified were distributed across 3 phyla, 11 classes, 29 orders, 39 families, and 51 genera. These phyla included Bacillariophyta (previously classified under Heterokontophyta), Chlorophyta, and Rhodophyta. Among them, Bacillariophyta and Chlorophyta were the dominant groups. It is noteworthy that Chlorophyta exhibited particularly high dominance in V1 (Figure 2a). The most abundant species monitored across ships included *Chlorella* sp. ind‐29, *Skeletonema marinoi*, *Leptocylindrus* sp. RCC4827, *Skeletonema* sp. RCC1866, *Chaetoceros* sp. 19 LG‐2014, and *Melosira moniliformis* (Figure 3a). Furthermore, community composition in vessels V1, V3, and V6 was highly skewed, with over 90% of total reads derived from only 2, 1, and 3 species, respectively.

A total of 70 invertebrate species were identified, spanning 15 phyla, 29 classes, 44 orders, 64 families, and 66 genera. The phyla Discosea, Arthropoda, Ciliophora, and Porifera were dominant in terms of taxonomic representation (Figure 2b). The most abundant invertebrate taxa across the surveyed vessels included *Pseudoparamoeba* sp., *Tetmemena* sp. Y1, *Paracalanus* sp. Roadstead of Brest, *Paramecium caudatum* , *Dysidea* sp. QMG318930, and *Euplotes* sp. MdV3B (Figure 3b). Notably, *Pseudoparamoeba* sp. displayed overwhelming dominance in V1, accounting for 91% of the total relative abundance.

A total of 100 fish species were identified, encompassing 2 classes, 36 orders, 64 families, and 90 genera. The dominant genera included Cryptopsaras, Anguilla, and Regalecus (Figure 2c). The relative abundance profiles indicated that the principal species monitored across vessels were *Cryptopsaras couesii* , *Anguilla anguilla* , and *Regalecus glesne* (Figure 3c). In all ships except V3 and V6, more than 90% of the total abundance was dominated by no more than three species. Especially, *R. glesne* accounted for the highest proportion in ships V3, V4, V5, and V6.

### 3.2 Results of Species Diversity Analysis

Rarefaction curve analyses demonstrated that most samples approached or attained a stable plateau, indicating that the sequencing depth was generally sufficient to capture the majority of community‐level species diversity (Figures S1 and S2). However, the phytoplankton assemblage from V4 did not exhibit a saturation plateau, suggesting that additional taxa may remain undetected at the current sequencing depth.

As summarized in Table 3, α‐diversity indices exhibited marked variation among vessels for phytoplankton, invertebrates, and fish. For phytoplankton, V6 displayed the greatest species richness (Chao1 = 35), whereas V2 recorded the highest Shannon index (2.119). In contrast, V4 exhibited the highest Pielou index (0.855) despite its low richness (Chao1 = 9). For invertebrates, V6 again showed the highest richness (Chao1 = 38), while V2 achieved the highest diversity and evenness (Shannon = 2.363; Pielou = 0.734). For fishes, the greatest diversity was monitored in V3 (Chao1 = 64.75; Shannon = 1.918; Pielou = 0.461).

**TABLE 3:** Alpha diversity analysis of ballast water across different ships.

	Diversity index	V1	V2	V3	V4	V5	V6
Phytoplankton	Chao1	23	31	27	9	44	35
	Shannon	0.809498	2.118563	0.349149	1.878037	1.398907	0.711389
	Pielou	0.258172	0.61694	0.105936	0.854732	0.369671	0.20009
Invertebrate	Chao1	18	25	31	10	17	38
	Shannon	0.391069	2.363418	1.956468	1.476525	1.287321	1.474188
	Pielou	0.135301	0.734237	0.569737	0.641247	0.454368	0.405265
Fish	Chao1	17	10	64.75	39	18	50
	Shannon	0.026276	0.350567	1.91789	0.317849	0.190853	1.335052
	Pielou	0.009274	0.152249	0.461155	0.08676	0.066031	0.341269

The α‐diversity indices for individual ballast tank samples are summarized in Table 4. Within V5, sample V52 exhibited the highest phytoplankton richness (Chao1 = 39), diversity (Shannon = 1.478), and evenness (Pielou = 0.404). For invertebrates, V52 likewise recorded the greatest richness and diversity (Chao1 = 13; Shannon = 1.256), although sample V51 displayed higher evenness (Pielou = 0.512). With respect to fishes, V52 showed greater diversity indices than V51 (Chao1 = 16; Shannon = 0.227; Pielou = 0.082). In V6, sample V63 contained the most diverse phytoplankton assemblage (Chao1 = 26; Shannon = 0.796; Pielou = 0.244). For invertebrates, V63 exhibited the highest richness (Chao1 = 28), whereas V61 displayed the greatest diversity (Shannon = 1.863) and evenness (Pielou = 0.612). Among fish, V63 presented the highest Shannon (1.750) and Pielou (0.505) indices, although species richness was greatest in V61 (Chao1 = 50).

**TABLE 4:** Alpha diversity analysis of ballast water in different tanks.

	Diversity index	V51	V52	V61	V62	V63
Phytoplankton	Chao1	35	39	18	19	26
	Shannon	1.317661	1.478342	0.617593	0.715565	0.796
	Pielou	0.370614	0.403526	0.213672	0.243022	0.244315
Invertebrate	Chao1	11	13	21	17	28
	Shannon	1.228029	1.256233	1.863182	0.853193	1.445205
	Pielou	0.512128	0.489769	0.611978	0.30114	0.433708
Fish	Chao1	10	16	50	26	32.333333
	Shannon	0.115216	0.226853	0.354687	0.435151	1.750202
	Pielou	0.050038	0.08182	0.099761	0.13356	0.505002

**FIGURE 4:**
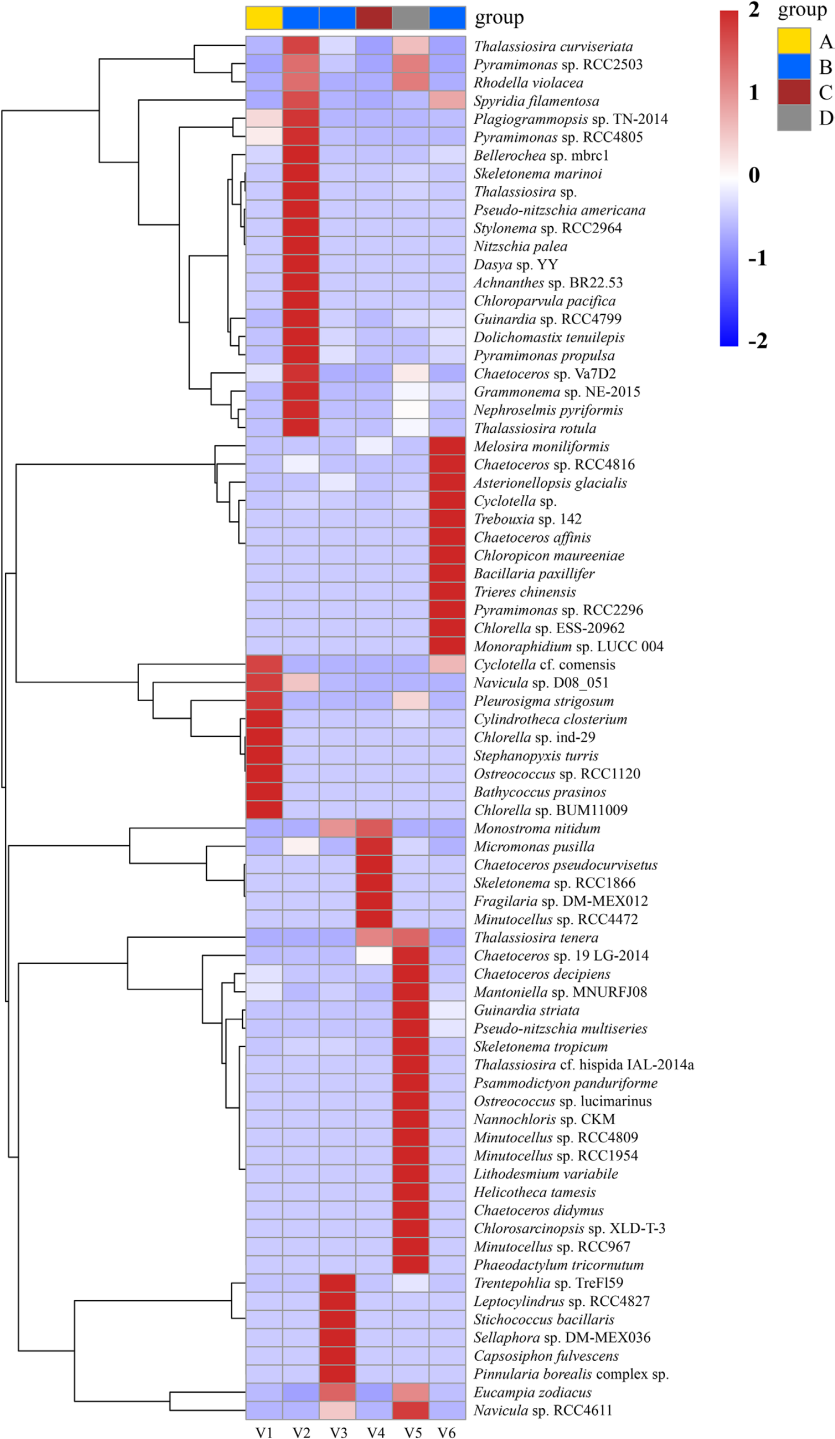
The heatmap illustrates the differences in phytoplankton assemblage structure across different sea areas. Samples are grouped based on their geographical origin. Values range from −2 to 2, representing *z*‐score standardized species abundance: 0 indicates average abundance, negative values indicate below‐average abundance, and positive values indicate above‐average abundance.

**FIGURE 5:**
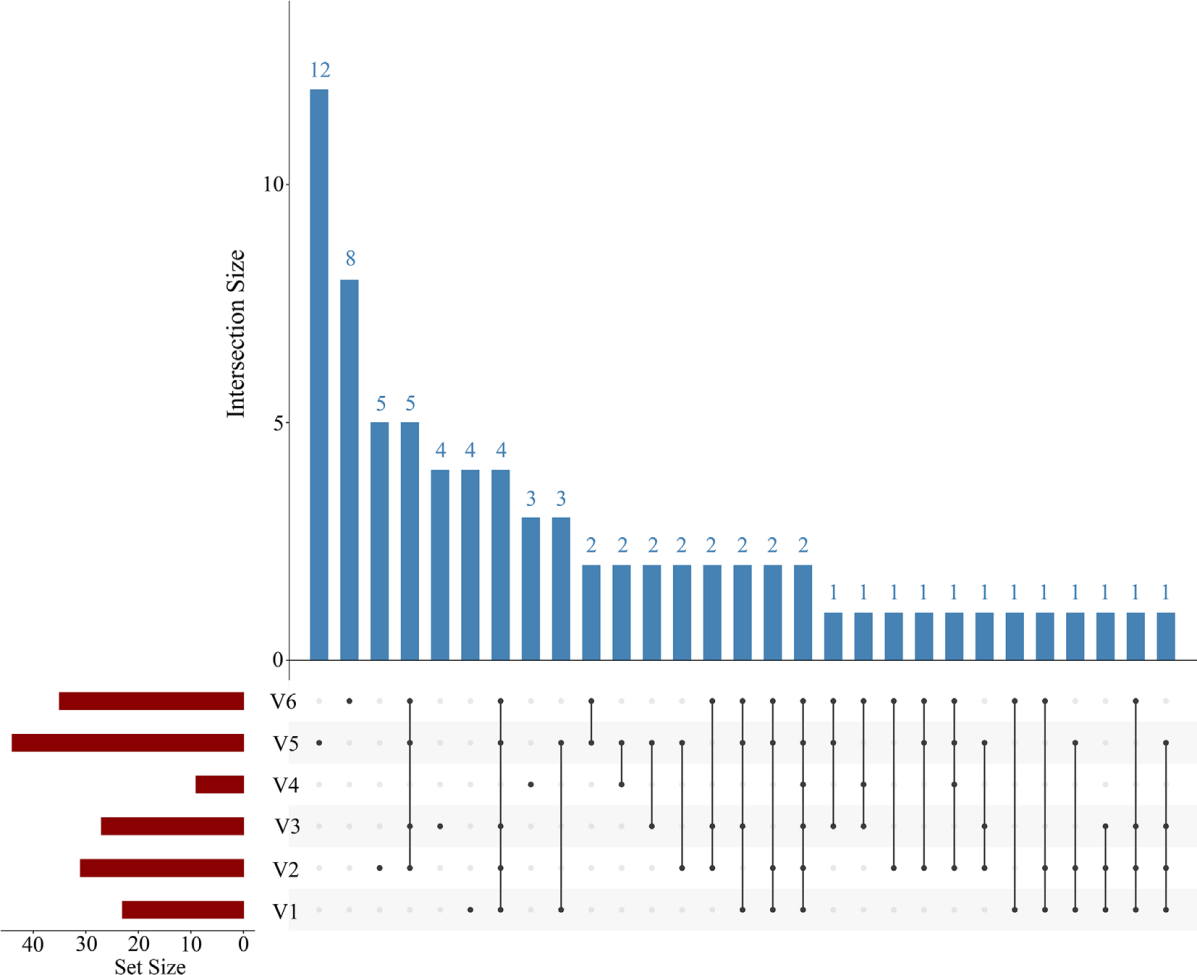
UpSet plot of phytoplankton species in different ships. The red bars represent the number of species in each sample, the black dots in the matrix represent individual samples, the connected black dots indicate shared species, and the blue bars represent the corresponding number of species.

Heatmaps and UpSet plots further illustrate the differences in species composition among ships and ballast tanks. As shown in Figure 4, the heatmap revealed pronounced dissimilarities in phytoplankton assemblages across vessels. For instance, although vessels V2, V3, and V6 all originated from Seto Inland Sea, their species profiles differed substantially, suggesting that factors beyond ballast water source may strongly influence community structure. Similarly, V3 and V6, which had comparable ballast water uptake locations and ages, nonetheless displayed striking compositional divergence. *Leptocylindrus* sp. RCC4827 was dominant in V3 but absent from V6, whereas *Capsosiphon fulvescens* , *Navicula* sp. D08_051, *Trentepohlia* sp. TreFl59, *Sellaphora* sp. DM‐MEX036, *Thalassiosira rotula* , *Pinnularia borealis* complex sp., *Stichococcus bacillaris* , *Navicula* sp. RCC4611, and *Pyramimonas* sp. RCC4805 were exclusively monitored in V3. The UpSet plot further supports these findings. Among the 76 phytoplankton species identified, only two— *Micromonas pusilla* and *Melosira moniliformis* —were shared across all six vessels (Figure 5). Each ship also harbored unique species not found in the others; notably, V5 contained the highest number of unique species (*n* = 12), followed by V6 (*n* = 8). Even V4, which had the lowest overall species richness, contained three unique species. Further analysis of individual ballast tanks within ships V5 and V6 (Figures S3 and S4) revealed that, while several species were shared among tanks—30 in V5 and 11 in V6—each tank within the same ship also contained unique taxa: V51 (*n* = 5), V52 (*n* = 9), V61 (*n* = 4), V62 (*n* = 3), and V63 (*n* = 11).

**FIGURE 6:**
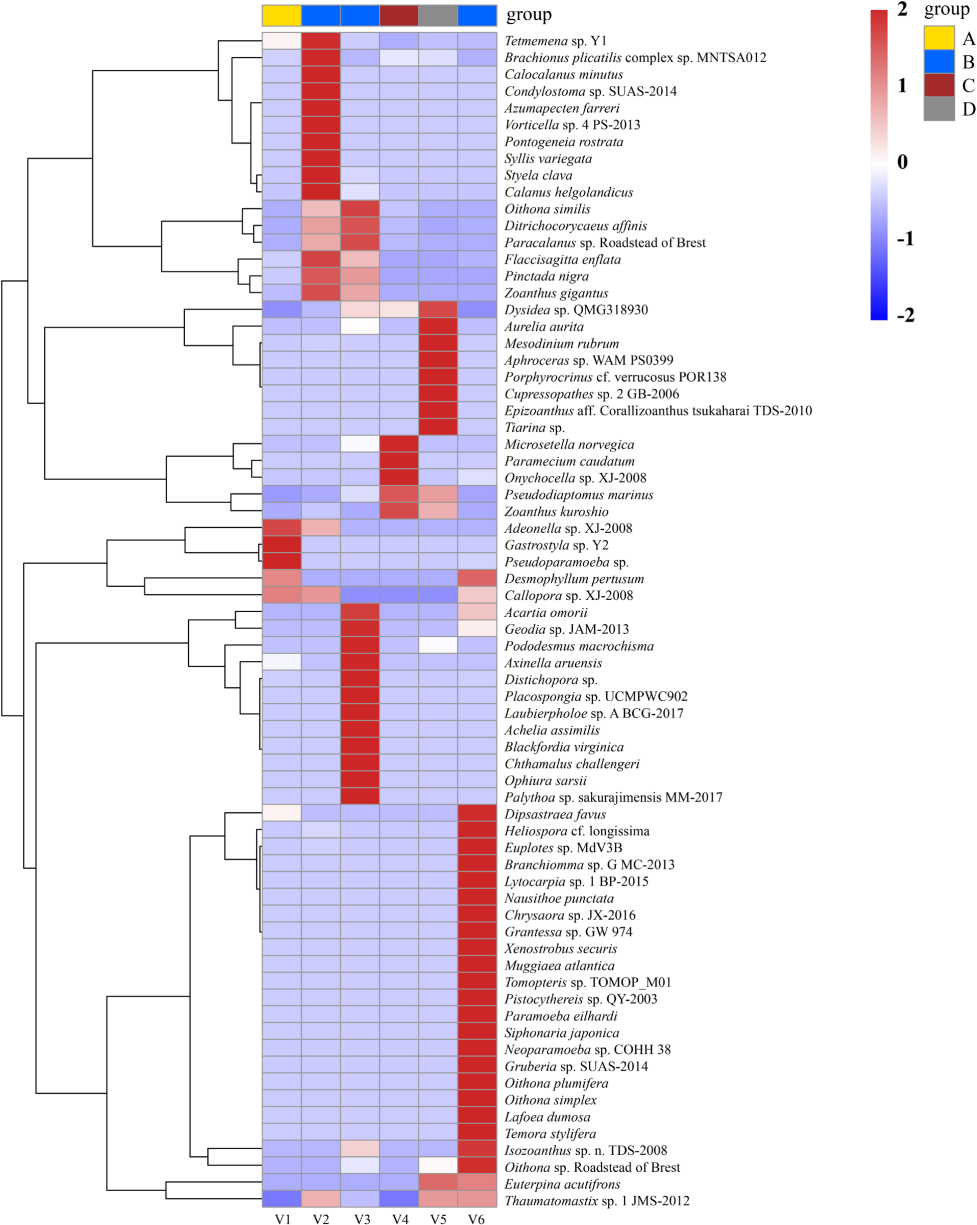
The heatmap illustrates the differences in invertebrate assemblage structure across different sea areas. Samples are grouped based on their geographical origin. Values range from −2 to 2, representing *z*‐score standardized species abundance: 0 indicates average abundance, negative values indicate below‐average abundance, and positive values indicate above‐average abundance.

**FIGURE 7:**
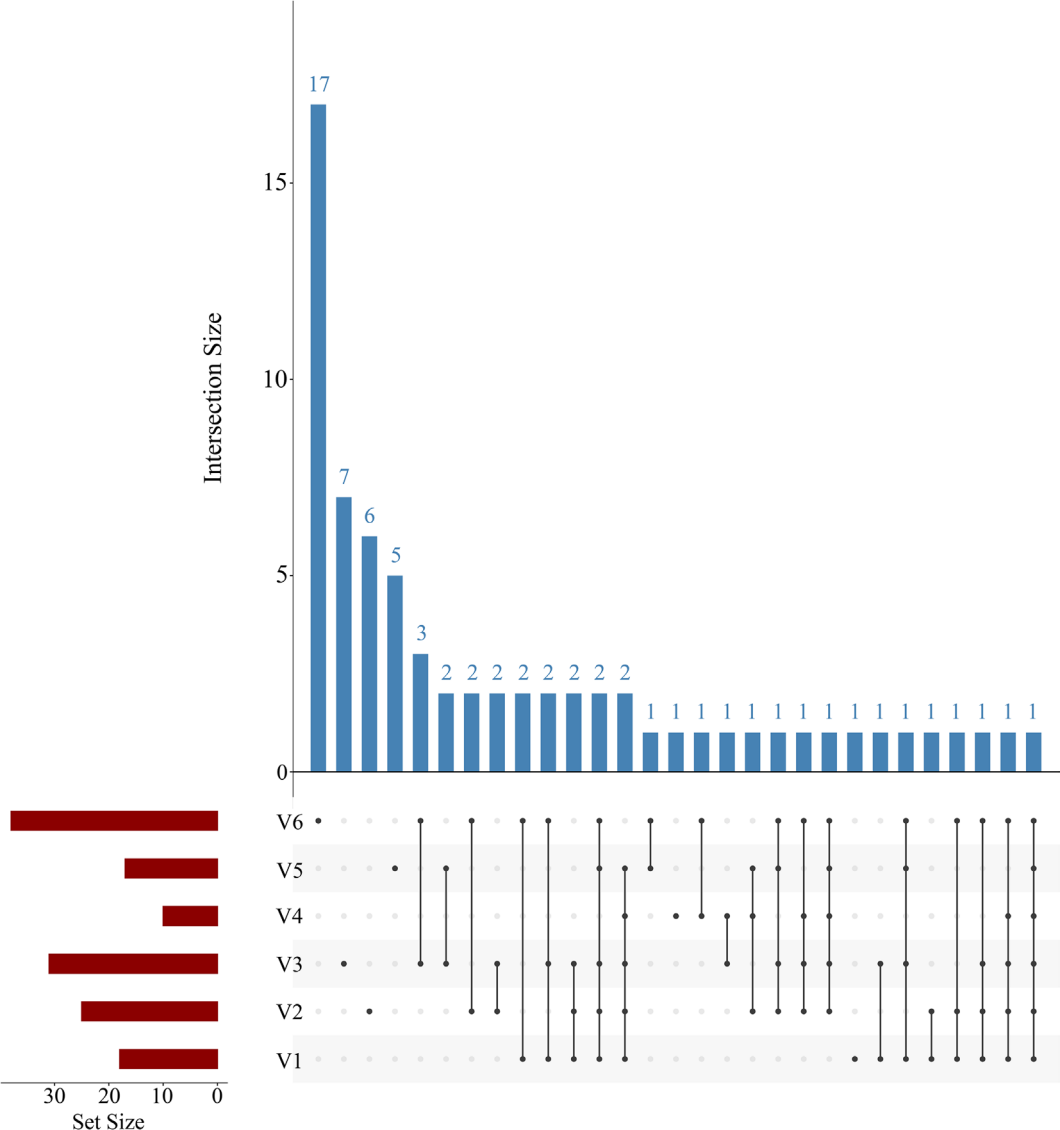
UpSet plot of invertebrate species in different ships. The red bars represent the number of species in each sample, the black dots in the matrix represent individual samples, the connected black dots indicate shared species, and the blue bars represent the corresponding number of species.

The invertebrate heatmap revealed partial overlap in species composition among ships; however, as with phytoplankton, assemblages also displayed pronounced inter‐ship differences (Figure 6). For example, although vessels V3 and V6 shared similar ballast water uptake locations and ages, their invertebrate communities were markedly distinct. In V3, *Paracalanus* sp. Roadstead of Brest and *Dysidea* sp. QMG318930 dominated, whereas *Euplotes* sp. MdV3Bs was most abundant in V6. Moreover, several species—including *Paracalanus* sp. Roadstead of Brest, *Zoanthus gigantus*, *Microsetella norvegica* , *Calanus helgolandicus* , *Ophiura sarsii*, *Pododesmus macrochisma* , *Laubierpholoe* sp. A BCG‐2017, *Pinctada nigra*, *Palythoa* sp. sakurajimensis MM‐2017, *Blackfordia virginica* , *Placospongia* sp. UCMPWC902, *Axinella aruensis*, *Achelia assimilis* , *Chthamalus challengeri*, *Brachionus plicatilis* complex sp. MNTSA012, *Aurelia aurita* , and *Styela clava—were* monitored exclusively in V3 and absent from V6. The UpSet plot further supports these findings. Among all six vessels, only one species (*Dysidea* sp. QMG318930) was shared universally, while each vessel harbored unique taxa. V6 contained the largest number of unique species (*n* = 17), followed by V3 (*n* = 7), V2 (*n* = 6), and V5 (*n* = 5); V1 and V4 each contained one unique species (Figure 7). Analysis at the level of individual ballast tanks (Figures S5 and S6) revealed that intra‐ship overlap was generally greater than inter‐ship overlap; nevertheless, distinct taxa were also monitored across tanks within the same vessel. For example, the two ballast tanks in V5 shared seven species, while V51 and V52 harbored two and six unique species, respectively. In V6, 10 species were shared across tanks, yet additional unique taxa were identified in V61 (*n* = 4), V62 (*n* = 4), and V63 (*n* = 12).

**FIGURE 8:**
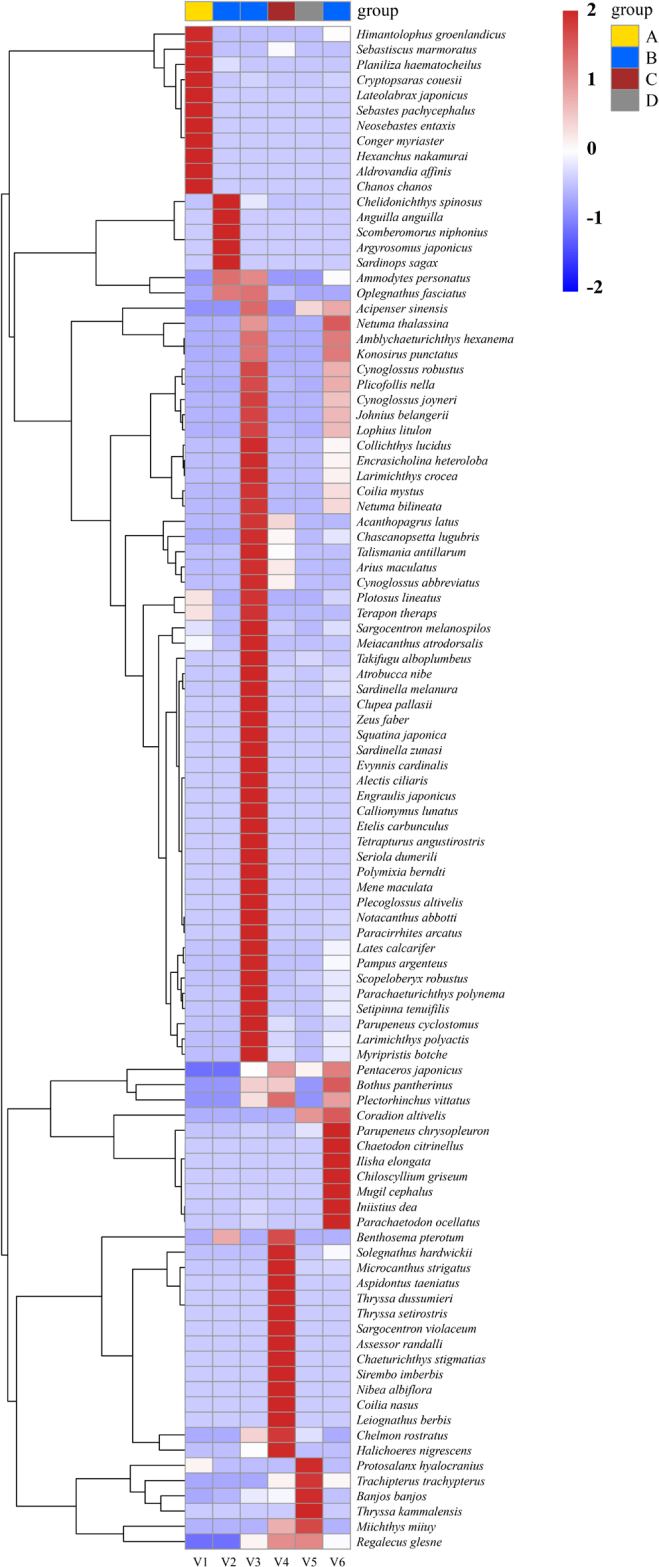
The heatmap illustrates the differences in fish assemblage structure across different sea areas. Samples are grouped based on their geographical origin. Values range from −2 to 2, representing *z*‐score standardized species abundance: 0 indicates average abundance, negative values indicate below‐average abundance, and positive values indicate above‐average abundance.

**FIGURE 9:**
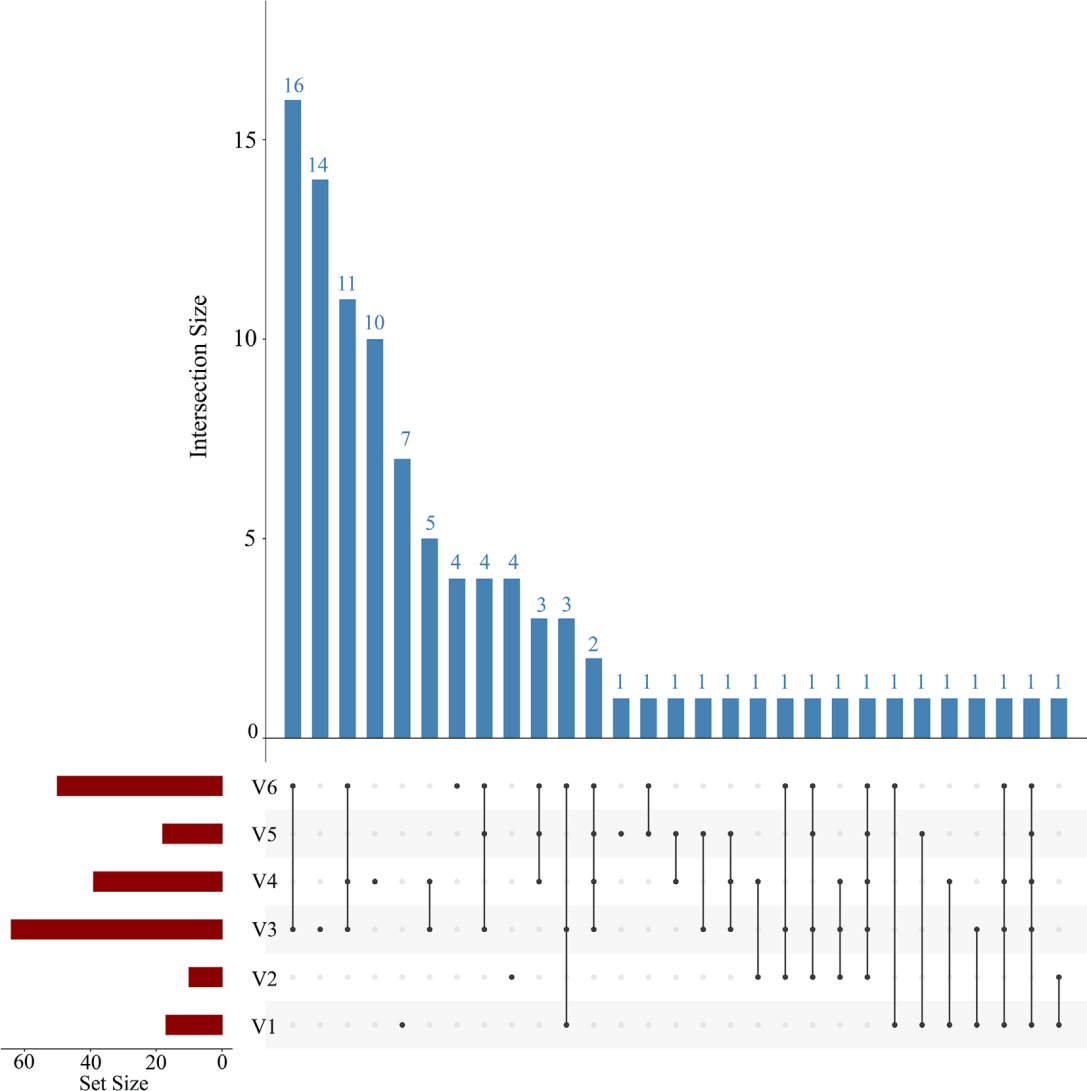
UpSet plot of fish species in different ships. The red bars represent the number of species in each sample, the black dots in the matrix represent individual samples, the connected black dots indicate shared species, and the blue bars represent the corresponding number of species.

Fish assemblages also exhibited pronounced variation among vessels. No single fish species was shared across all six ships, and only three species— *Chelidonichthys spinosus* , *Banjos banjos* , and *Ammodytes personatus* —were monitored in common among vessels V2, V3, and V6 (Figure 8). Notably, a high degree of similarity was observed between V3 and V6, which shared as many as 41 species (Figure 9). Nevertheless, each ship harbored distinct fish assemblages. V3 contained the largest number of unique species (*n* = 14), followed by V4 (*n* = 10), V1 (*n* = 7), V6 (*n* = 4), V2 (*n* = 4), and V5 (*n* = 1) (Figure 9). Analysis of ballast tanks within individual vessels revealed substantial intra‐ship variability in fish community composition (Figures S7 and S8). In V5, the two ballast tanks shared eight species, while V51 and V52 contained two and eight unique species, respectively. In V6, fifteen species were shared across all three tanks, yet each tank also harbored unique taxa: V61 (*n* = 10), V62 (*n* = 3), and V63 (*n* = 9).

**FIGURE 10:**
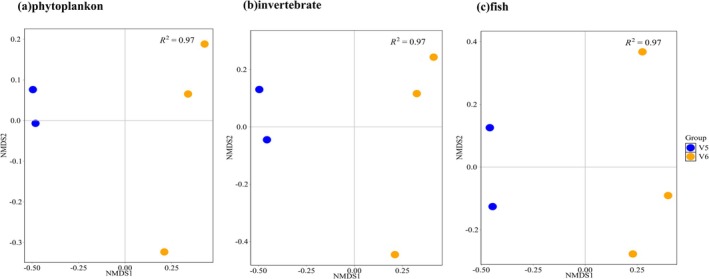
NMDS plots of phytoplankton, invertebrate, and fish groups in ballast tanks.

PERMANOVA analysis indicated that neither ballast water age nor ballast location had a statistically significant effect on the species composition of phytoplankton, invertebrates, or fish (*p* > 0.05; Table S3). Nevertheless, ballast location exerted a comparatively greater influence than ballast water age. For example, in phytoplankton, ballast location explained 59.0% of the variation in community composition, whereas ballast water age accounted for only 14.5%. NMDS ordination revealed clear differences in species composition among ballast tanks across all three taxonomic groups—phytoplankton, invertebrates, and fish (Figure 10).

These results demonstrate pronounced differences in species diversity and composition of phytoplankton, invertebrates, and fish among ships and across their ballast tanks. Notably, substantial heterogeneity was observed even among samples with comparable ballast water age and uptake location, indicating that additional factors may drive variation in community structure.

Additionally, the UpSet maps of different ballast tanks (Figures S4, S6, and S8) reveal that each ballast tank contained a unique subset of species. Although sampling from a single ballast tank generally captured the majority of species present within a ship, certain taxa were restricted to specific tanks and would remain undetected without broader sampling coverage.

### 3.3 Monitoring Results of Alien Species

Among the 76 phytoplankton species identified, 16 were identified as alien species (Table 5) (Lei 2021; Liu et al. 2014; Yin et al. 2010; Zu et al. 2000), none of which were listed in *China's Invasive Alien Species* (Xu and Qiang 2018). For invertebrates, 13 of the 70 identified species were recognized as alien, including *Styela clava* , which is recorded as an invasive species in *China's Invasive Alien Species* (Xu and Qiang 2018). Among the 100 fish species, 12 were identified as alien species, with *A. anguilla* and *Lates calcarifer* recognized as invasive taxa.

**TABLE 5:** Diversity of alien species in phytoplankton, invertebrates, and fish with literature records from the Qingdao area (invasive species in bold).

Category	Alien species	Any record in Qingdao
Phytoplankton	*Bathycoccus prasinos*	No
	*Micromonas pusilla*	Yes (Yin et al. 2010)
	*Skeletonema marinoi*	Yes (Lei 2021)
	*Thalassiosira curviseriata*	No
	*Spyridia filamentosa*	No
	*Trieres chinensis*	No
	*Pyramimonas propulsa*	No
	*Pseudo‐nitzschia americana*	No
	*Dolichomastix tenuilepis*	No
	*Chloropicon maureeniae*	No
	*Asterionellopsis glacialis*	No
	*Rhodella violacea*	No
	*Psammodictyon panduriforme*	No
	*Nephroselmis pyriformis*	No
	*Helicotheca tamesis*	No
	*Chloroparvula pacifica*	No
Invertebrata	*Calanus helgolandicus*	No
	*Zoanthus kuroshio*	No
	*Zoanthus gigantus*	No
	*Paramecium caudatum*	No
	*Desmophyllum pertusum*	No
	*Paramoeba eilhardi*	No
	*Mesodinium rubrum*	Yes (Liu et al. 2014)
	*Pododesmus macrochisma*	No
	*Xenostrobus securis*	No
	*Axinella aruensis*	No
	*Calocalanus minutus*	No
	*Dipsastraea favus*	No
	***Styela clava***	Yes (Zu et al. 2000)
Fish	*Regalecus glesne*	No
	*Netuma thalassina*	No
	*Encrasicholina heteroloba*	No
	***Lates calcarifer***	No
	*Netuma bilineata*	No
	*Solegnathus hardwickii*	No
	*Trachipterus trachypterus*	No
	*Himantolophus groenlandicus*	No
	*Talismania antillarum*	No
	*Sargocentron violaceum*	No
	***Anguilla anguilla***	No
	*Planiliza haematocheilus*	No

The two alien phytoplankton species, *Micromonas pusilla* and *S. marinoi*, have been previously recorded in the Qingdao area (Lei 2021; Yin et al. 2010). Similarly, the alien invertebrate *Mesodinium rubrum* and the invasive tunicate *S. clava* have been previously reported in Qingdao (Liu et al. 2014; Zu et al. 2000). In contrast, none of the alien fish species monitored in ballast water has yet been documented in the literature for this region.

The occurrence of alien species varied among ships (Table S4). For phytoplankton, ship V2 harbored the highest number of alien species (11), whereas ship V4 contained the fewest (1). Among invertebrates, ships V2 and V3 both recorded the highest number of alien taxa (6 each), including the invasive *S. clava*, while V4 contained only two alien invertebrate species. For fish, ship V6 exhibited the greatest number of alien species (8), whereas V1 and V2 contained the fewest (2 each). Notably, genetic evidence of the invasive *A. anguilla* was monitored in V2, while *L. calcarifer* was identified in the V3 and V6 ships.

## 4 Discussion

### 4.1 Effectiveness of eDNA Metabarcoding for Alien Species Monitoring

In this study, eDNA metabarcoding was applied to analyze ballast water samples from multiple ships. To ensure spatial relevance, species not reported at the ballast water uptake locations were excluded during data filtering, as described in the Methods section. Following this refinement, the majority of species retained were consistent with the known geographic ranges of their respective uptake sites. Only a small number of taxa were removed due to biogeographic inconsistencies, which could reflect potential contamination, taxonomic misassignments, or rare long‐distance dispersal events (Deiner et al. 2017). Overall, these findings demonstrate the accuracy and reliability of eDNA metabarcoding for monitoring species diversity. A key advantage of this approach lies in its high sensitivity, enabling the detection of trace amounts of genetic material and facilitating the identification of rare or cryptic species that may be overlooked by traditional survey methods (Adams et al. 2009; Fonseca et al. 2023).

However, we acknowledge that eDNA technology has certain limitations, particularly the potential for false positives (Goldberg et al. 2016). To address this, we implemented rigorous quality control protocols, including the use of negative controls and stringent sequence filtering. Sequences suspected of contamination were excluded to ensure that the results accurately reflected the species present in the ballast water samples. While eDNA may not capture the full spectrum of species diversity, it nonetheless provides a reliable and efficient approach for biodiversity monitoring in aquatic environments.

Moreover, eDNA metabarcoding is particularly advantageous for monitoring alien species in ballast water, where early monitoring is critical for mitigating both ecological and economic impacts. Its capacity to identify potential invasive species prior to their establishment enables proactive ecological management and informed policy development (Rishan et al. 2024). By bridging existing gaps in early monitoring and providing actionable biodiversity data, eDNA metabarcoding constitutes a valuable tool in global efforts to monitor and manage invasive species effectively.

In the present study, we successfully identified several alien species, including three recognized invasive species: *S. clava* , *A. anguilla* , and *L. calcarifer* . These species have been documented to cause severe ecological disruption and substantial economic losses in multiple regions (Arai et al. 2017; Çinar 2016; Pusey et al. 2006), thereby confirming that ballast water functions as an important vector for the interregional dispersal of alien taxa. This finding underscores the necessity of systematic monitoring and accurate taxonomic identification to support the implementation of effective control and management strategies.

Unlike many previous eDNA studies that primarily focused on a single taxonomic group (e.g., phytoplankton or bacteria), the present study applied three universal primer sets (18S V4, 18S V9, and 12S) to simultaneously target phytoplankton, invertebrates, and fish. This multi‐marker approach enabled a more comprehensive biodiversity assessment across multiple trophic levels while enhancing the capacity for alien species monitoring. To our knowledge, this represents the first application of eDNA metabarcoding for multi‐taxa ballast water monitoring at Dongjiakou Port. Given the current paucity of comparable studies in this region, direct cross‐study comparisons remain challenging. Future investigations that adopt standardized sampling protocols and primer strategies across ports will be crucial for establishing baselines, benchmarking biodiversity patterns, and assessing invasion risks on broader spatial scales.

### 4.2 Species Composition Characteristics and Factors Influencing Ballast Water Communities

In this study, we found that ballast water communities were typically characterized by the dominance of a limited number of species. Such skewed abundance distributions suggest pronounced dominance effects, with certain taxa exerting disproportionate influence on community structure. This prevalence likely reflects their ecological adaptability and potential competitive advantages within the confined and often stressed environmental conditions of ballast tanks. These patterns underscore the importance of considering not only species richness but also dominance dynamics when evaluating biodiversity in ballast water ecosystems.

Among the phytoplankton, two species— *M. moniliformis* and *Micromonas pusilla—*were consistently monitored across ballast water samples from all six ships. Both taxa are characterized by broad biogeographic distributions and high adaptability to fluctuating environmental conditions. For invertebrates, the only common species was *Dysidea* sp. QMG318930, which similarly exhibits pronounced ecological plasticity and resilience within ballast water habitats. In contrast, no single fish species was shared across all ships, reflecting the comparatively higher variability and potential environmental sensitivity of fish assemblages. Collectively, these findings suggest that ballast water communities tend to harbor species with broad ecological tolerances, likely due to their capacity to withstand the harsh and dynamic conditions within ballast tanks. Such adaptability increases the likelihood of successful establishment upon release into recipient environments, underscoring the importance of prioritizing the monitoring of taxa with wide ecological niches (Ardura, Martinez, et al. 2021).

The species composition of the three major taxonomic groups detected in ballast water—phytoplankton, invertebrates and fish—varied substantially across ship samples. This variability likely reflects, at least in part, that the vessels originated from diverse geographic regions spanning the South China Sea, Seto Inland Sea, Taiwan Strait and the Yellow Sea, and that the monitored species corresponded to ecological communities characteristic of these regions. Although previous studies have emphasized ballast location, ballast water age, and tank environment as important factors of community composition (Briski et al. 2012; Ghabooli et al. 2016; Yang et al. 2018), the PERMANOVA analysis in this study indicated that ballast water age and ballast location did not have significant effects (*p* > 0.05). This finding suggests that other factors—such as microenvironmental conditions within tanks, voyage duration or operational practices (e.g., ballast water exchange procedures)—may exert a more critical influence on shaping species diversity. The pronounced differences observed between the V3 and V6 samples, despite their shared ballast water age and source location, further support this interpretation.

The NMDS results revealed pronounced differences in species composition among ballast tanks within the same vessel (Figure 10). Such intra‐ship variability may be attributed to differences in species assemblages within ballast tank sediments (Shang et al. 2024). Operational disturbances, such as agitation during ship maneuvers, can resuspend sediment‐associated organisms into the water column, thereby influencing community composition (Darling et al. 2018). Previous studies have demonstrated that ballast tank sediments often contain abundant phytoplankton cysts and dormant invertebrates, which act as reservoirs for recolonization when environmental conditions become favorable (Maglić et al. 2017). This mechanism may explain why the V6 samples, derived from three ballast tanks, contained higher abundances of phytoplankton and invertebrates, whereas the V3 samples, collected from a single tank, showed comparatively lower abundance. These findings highlight the distinct biodiversity profiles of individual ballast tanks and underscore the importance of considering intra‐vessel variability. Consequently, we emphasize that sampling multiple ballast tanks within a ship is essential for obtaining a comprehensive assessment of ballast water biodiversity and for reducing the risk of underestimating species richness or overlooking ecologically important taxa when relying on single‐tank sampling.

Previous studies have demonstrated that salinity is a key driver of species composition and biodiversity in aquatic environments, particularly within microbial communities in ballast water (Lymperopoulou and Dobbs 2017). Similarly, carbon (C) and nitrogen (N) concentrations have been shown to significantly influence the structure of bacterial assemblages in ballast tank sediments (Lv et al. 2017). In the present study, however, critical environmental parameters such as temperature, salinity, and pH were not measured due to constraints in field sampling conditions and resource availability. This limitation restricts our ability to directly evaluate the influence of these variables on observed patterns of species diversity. To address this gap, future research should incorporate systematic measurements of environmental factors (e.g., temperature, salinity, pH) alongside eDNA sampling and integrate ballast tank–specific characteristics to achieve a more comprehensive understanding of their roles in shaping species community structure.

Other potential influences, such as specific ballast water treatment practices or microenvironmental variations across sampling sites, may also play a significant role in shaping species composition (Hess‐Erga et al. 2019; Rey et al. 2019). These underexplored drivers warrant further investigation to clarify their ecological impacts on ballast water biodiversity. Future research should therefore prioritize the optimization of sampling methods and monitoring protocols to include a wider suite of environmental parameters, such as temperature, pH, and salinity. Broadening the scope of inquiry will not only deepen our understanding of the ecological dynamics within ballast water but also strengthen the scientific foundation for developing effective risk management and prevention strategies. Such advancements are essential to support global initiatives aimed at monitoring and controlling biological invasions.

### 4.3 Alien Species Risks and High‐Risk Source Regions

In this study, we identified 16 alien phytoplankton species, 13 alien invertebrate species, and 12 alien fish species. Distribution patterns across different ships and ballast water sources (Table S4, Figure 1) revealed notably higher alien species diversity in the V2, V3, and V6 samples. Importantly, all three invasive species detected in this study— *A. anguilla* , *L. calcarifer* , and *S. clava* —were found in samples from these same ships. These results suggest that vessels originating from Seto Inland Sea present a disproportionately higher risk of transporting alien and invasive species compared with those from other regions. This elevated risk may stem from the high native biodiversity in Seto Inland Sea or from environmental and operational conditions that enhance species survival and transport. Collectively, these findings highlight the need for targeted monitoring and stricter management of vessels from high‐risk regions to reduce the likelihood of alien species introductions.

In addition, the presence of certain alien species at high abundance levels across multiple ships raises particular concern. For instance, the fish *R. glesne* was dominant in the ballast water of ships V3, V4, V5, and V6. Its frequent occurrence across different vessels indicates an elevated risk of invasion through repeated introductions. Previous research has shown that species consistently detected in multiple samples have a greater likelihood of establishment and proliferation in new environments (Pagenkopp Lohan et al. 2016). This underscores the need to prioritize monitoring of species that are both abundant and frequently transported, as they represent critical targets for invasive species management. Moreover, alien species already monitored in Qingdao, such as *M. pusilla* , *S. marinoi*, *M. rubrum* , and *S. clava* (invasive species), warrant close attention. Continuous introductions of these taxa may facilitate their transition into invasive species, with potentially severe ecological, economic, and societal impacts.

Three invasive species were identified in this study: *S. clava* (detected in V2 and V3), *A. anguilla* (in V2), and *L. calcarifer* (in V3 and V6). Each of these taxa poses substantial ecological and economic threats. *S. clava* significantly disrupts native benthic communities by displacing dominant taxa and poses a serious threat to shellfish aquaculture (Çinar 2016). In Canada, its spread has resulted in annual economic losses to the shellfish industry estimated between $3.4 and $8.8 billion (Colautti et al. 2006). *A. anguilla* , originally introduced to Japan, is capable of long‐term survival, with monitoring records confirming persistence of mature individuals for more than a decade (Arai et al. 2017). As a top predator, it competes with the native *A. japonica* for resources and may introduce parasites that endanger local populations. Moreover, interbreeding between the two species can cause genetic introgression, potentially leading to irreversible impacts on ecosystem integrity (Arai et al. 2017). *L. calcarifer* , native to Australia, has demonstrated strong invasive potential and has been repeatedly recorded in artificial lagoons at the northern Red Sea (Stern and Rothman 2021). This species preys on local fish, shrimp, crabs, mollusks, and worms, thereby intensifying competition with native fishes for ecological niches and suppressing their growth and survival (Pusey et al. 2006). Collectively, these three invasive species pose significant ecological and economic threats, underscoring the need for rigorous monitoring and management.

Moreover, compared with the other invasive species identified in this study, the markedly high abundance of *A. anguilla* suggests an increased likelihood of successful population establishment, thereby posing a heightened invasion risk. This finding underscores the imperative of implementing targeted management strategies for high‐risk alien species to prevent their establishment and spread via ballast water, thereby mitigating the consequent adverse impacts on ecosystem functioning.

### 4.4 Implications for Ballast Water Management

The findings of this study underscore the challenges of ballast water management, particularly the complexity introduced by ship‐specific and tank‐specific variation. While ballast location and water age did not significantly influence species composition, characteristics unique to individual ballast tanks played an important role in shaping species diversity and distribution. Effective mitigation of alien species introductions, therefore, requires a comprehensive monitoring and management framework. Monitoring should involve sampling across multiple ballast tanks within the same ship, rather than relying on a single tank, to ensure an accurate assessment of overall invasion risk. From a management perspective, priority should be given to high‐risk ballast water sources, with targeted interventions such as stricter treatment protocols, more frequent water exchanges, and routine inspection of tanks with known contamination histories. Adoption of these tailored strategies would substantially improve ballast water management effectiveness and reduce the probability of alien species establishment.

## 5 Conclusion

This study represents the first application of eDNA metabarcoding in China to assess species diversity and alien species composition in ballast water at Dongjiakou Port, spanning phytoplankton, invertebrates, and fish. The results demonstrate that while ballast location and water age did not significantly influence species composition, pronounced differences were observed among ballast tanks within the same ship. Genetic evidence of multiple alien species was monitored, including three recognized invasive species: *S. clava* , *L. calcarifer* , and *A. anguilla* , with *A. anguilla* exhibiting particularly high abundance and posing a considerable threat to the coastal ecosystems of Qingdao. Collectively, these findings highlight ballast water as a critical vector for the introduction of alien species and underscore the urgent need for robust monitoring and management strategies to mitigate ecological risks.

This research provides a critical scientific foundation for improving ballast water management, thereby strengthening biosecurity measures and supporting global initiatives to prevent the spread of invasive species. By advancing understanding of ballast water biodiversity and the mechanisms of species introduction, this study contributes to the development of sustainable practices aimed at safeguarding coastal ecosystems and preserving global biodiversity.

## Author Contributions

**Hanglei Li:** conceptualization (equal), data curation (equal), formal analysis (equal), investigation (equal), methodology (equal), software (equal), validation (equal), visualization (equal), writing – original draft (lead), writing – review and editing (lead). **Hui Jia:** conceptualization (equal), data curation (equal), formal analysis (equal), investigation (equal), methodology (equal), software (equal), validation (equal), writing – review and editing (supporting). **Jingbo Peng:** investigation (equal), methodology (equal), resources (equal). **Xiaofeng Peng:** investigation (equal), resources (equal). **Zhipeng Ren:** investigation (equal), resources (equal). **Hui Zhang:** conceptualization (equal), formal analysis (equal), funding acquisition (lead), methodology (equal), project administration (lead), resources (equal), supervision (equal), validation (equal), writing – review and editing (lead).

## Conflicts of Interest

The authors declare no conflicts of interest.

## Supporting information


**Appendix S1:** ece372320‐sup‐0001‐AppendixS1.docx.

## Data Availability

Sequencing data and metadata files can be accessed through the NCBI Sequence Read Archive (SRA) repository as follows: Genetic data: Raw sequence reads are deposited in the SRA (BioProject PRJNA1219795). The original data file for the article can be accessed with the following link: https://www.ncbi.nlm.nih.gov/sra/?term=PRJNA1219795.
