# Supplementary material for: Monitoring Alien Species Diversity in Ballast Water Based on Environmental DNA Metabarcoding
Source: Ecol Evol. 2025 Oct 14;15(10):e72320. doi: 10.1002/ece3.72320 (PMC12519623; doi:10.1002/ece3.72320)
Supplement: Supplementary file 1 — Appendix S1: ece372320‐sup‐0001‐AppendixS1.docx. [file ECE3-15-e72320-s001.docx]

# Appendix


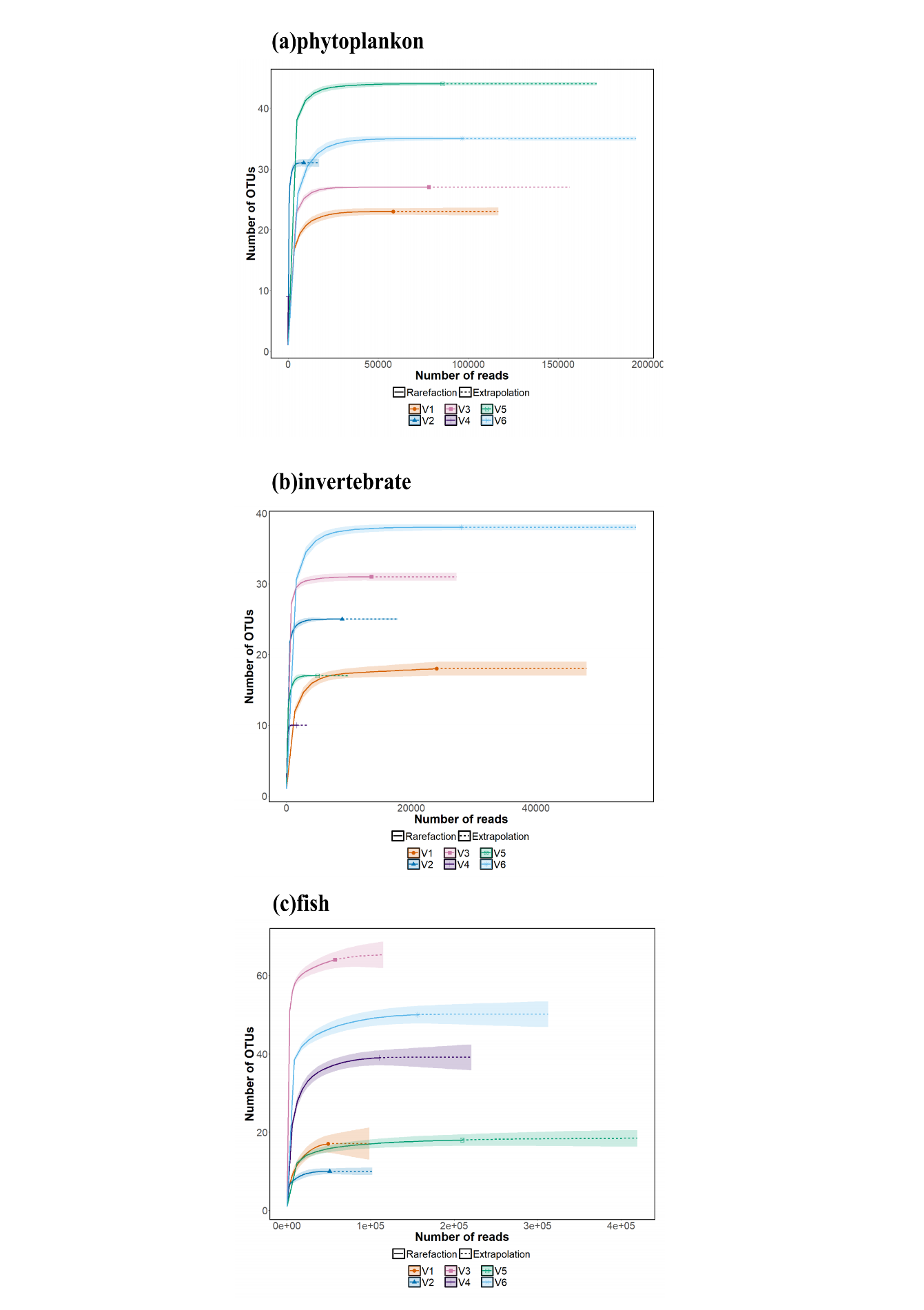


**Fig. S1.** Rarefaction and extrapolation curves for species richness (q = 0) across different ships. The curves are constructed by plotting the number of reads against the number of OTUs, with each line representing a different ship (V1–V6).


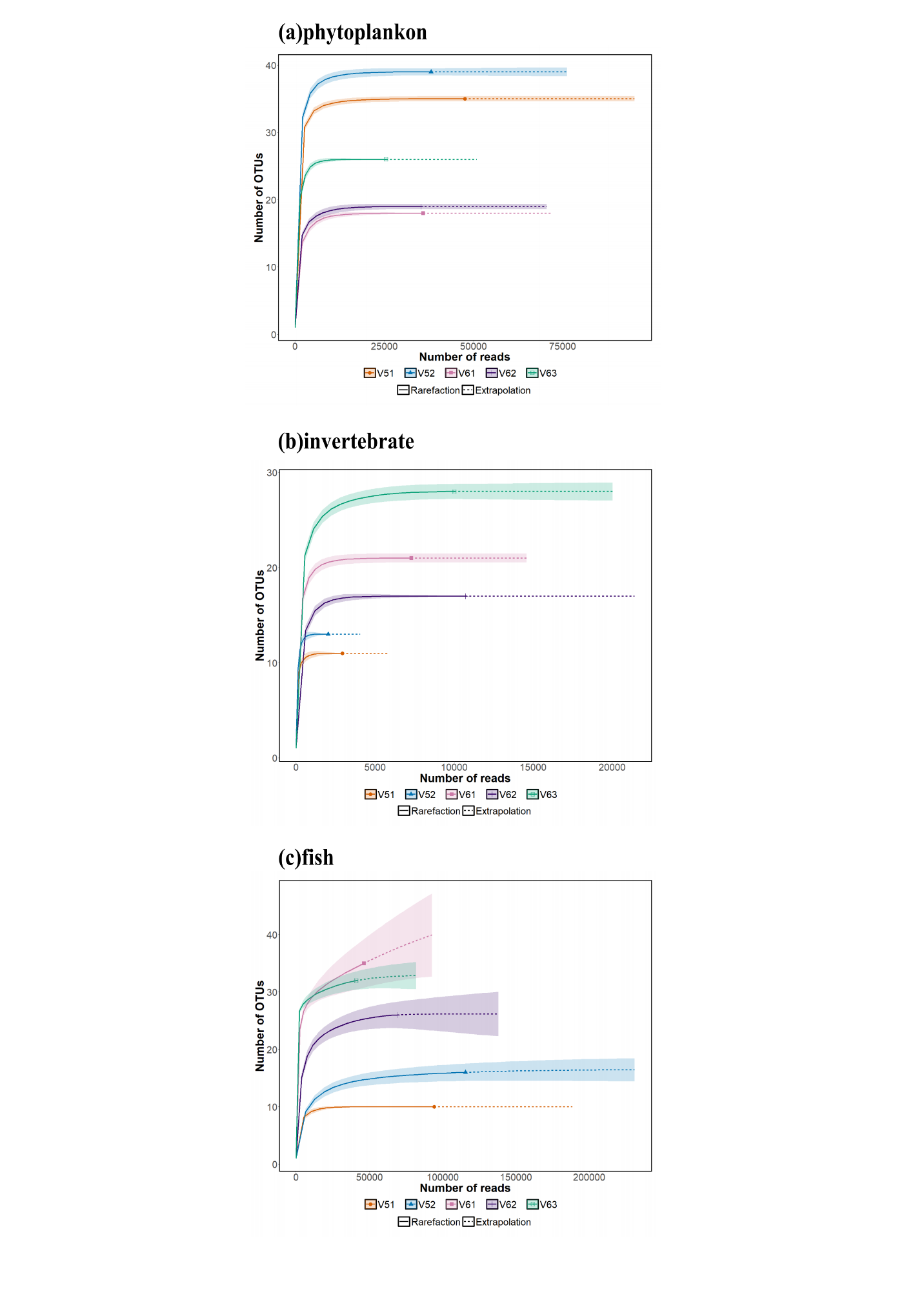


**Fig. S2.** Rarefaction and extrapolation curves for species richness (q = 0) across different ballast tanks. The curves plot the number of reads against the number of OTUs, with each line representing a different ballast tank (V51 - V52, V61 - V63).


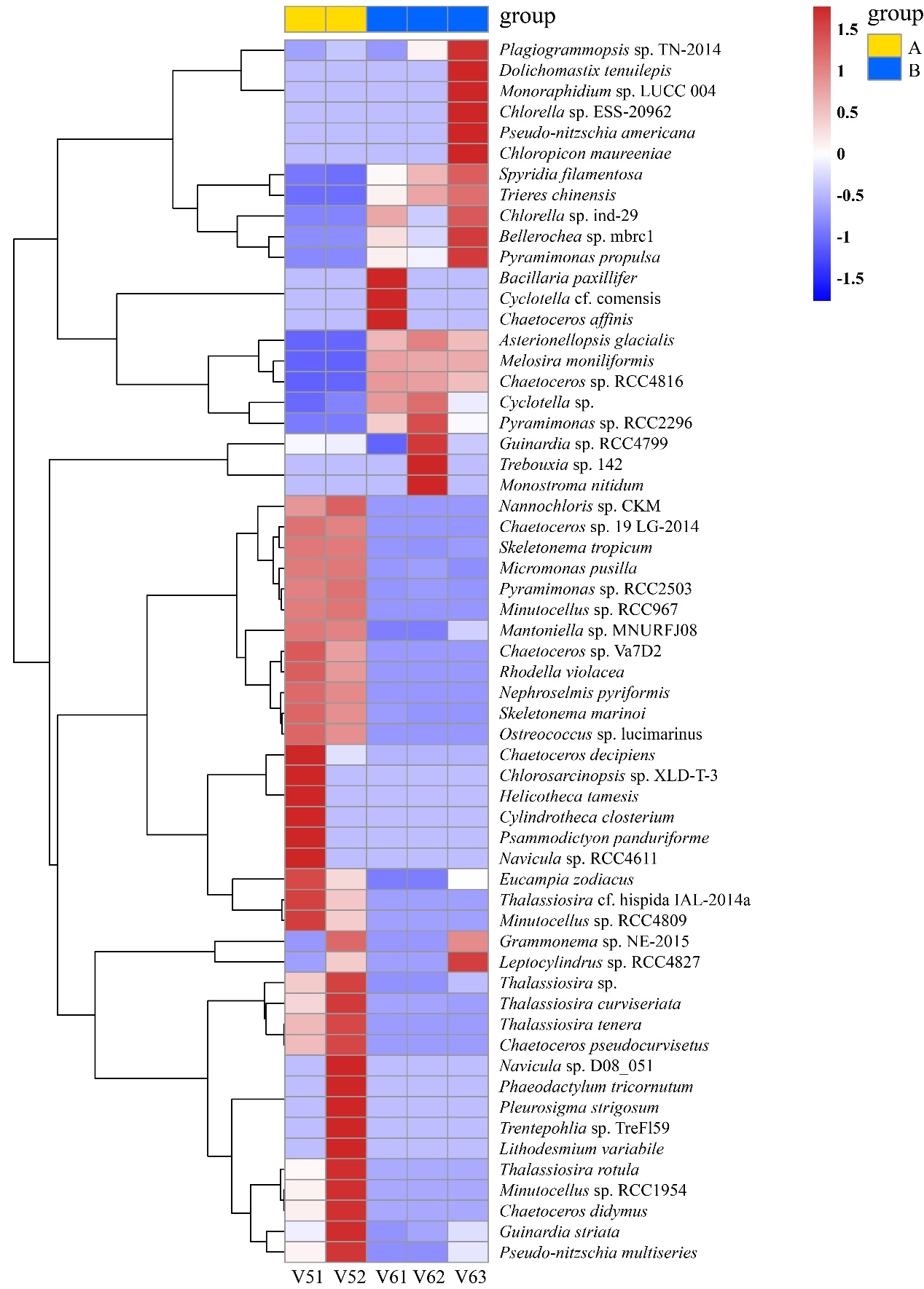


**Fig. S3.** The heatmap illustrates the differences in phytoplankton assemblage structure across different ballast tanks. Samples are grouped according to the origin of the ships. Values range from -1.5 to 1.5, representing z-score standardized species abundance: 0 indicates average abundance, negative values indicate below-average abundance, and positive values indicate above-average abundance.


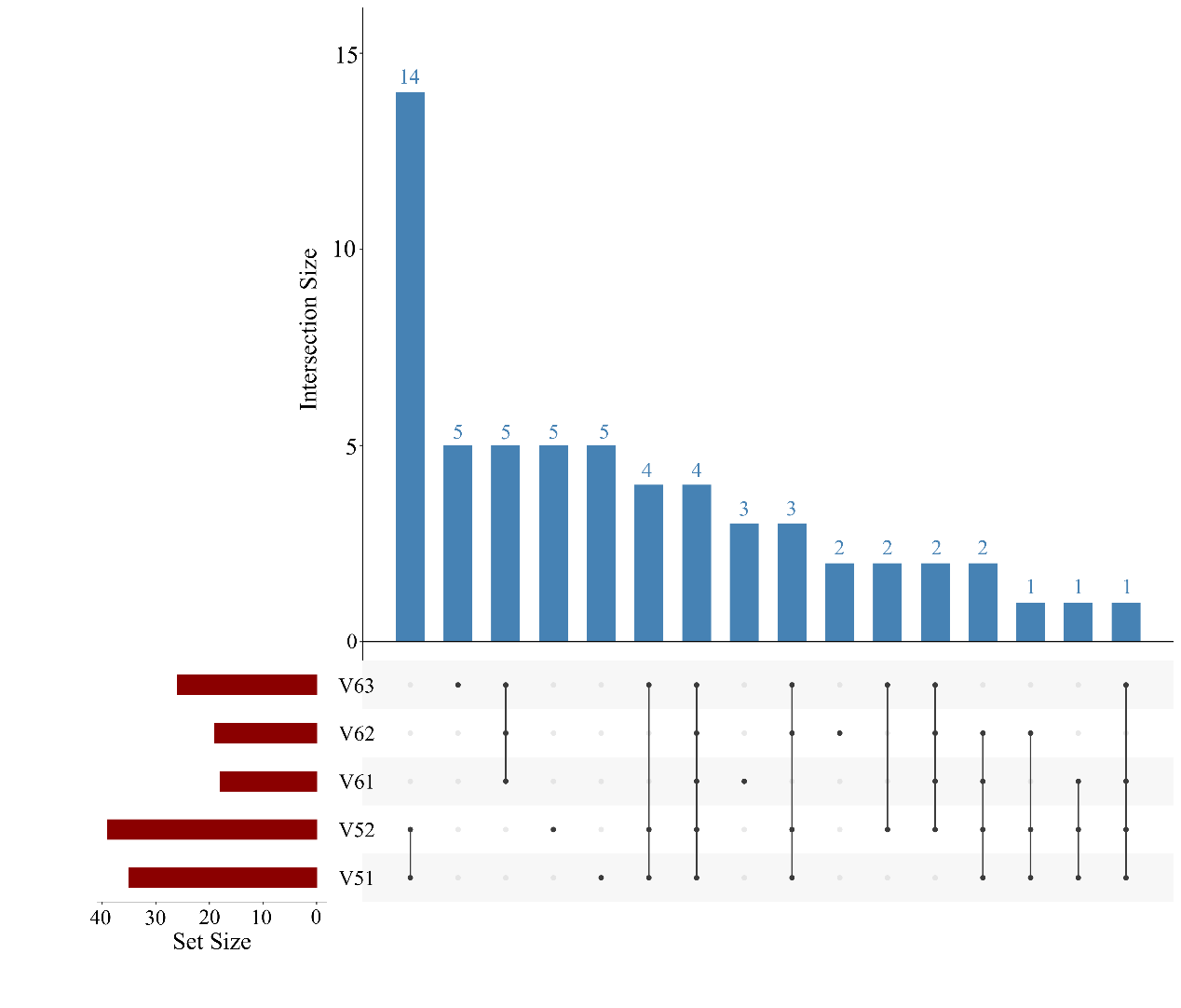


**Fig. S4.** UpSet plot of phytoplankton species in different ballast tanks. The red bars represent the number of species in each tank, while the black dots in the matrix indicate individual samples. Connected black dots represent shared species, and the blue bars show the corresponding number of species.


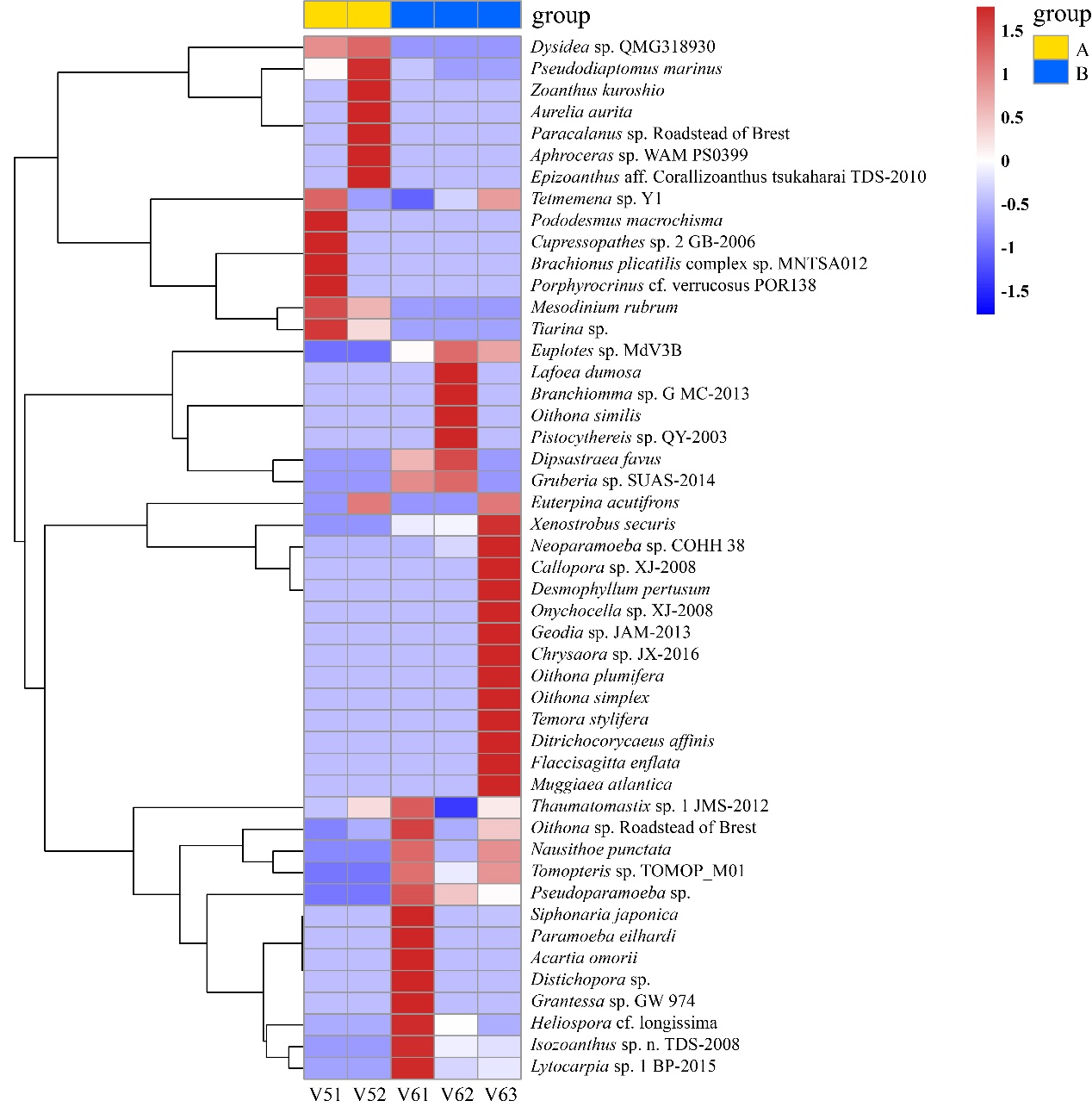


**Fig. S5.** The heatmap illustrates the differences in invertebrates assemblage structure across different ballast tanks. Samples are grouped according to the origin of the ships. Values range from -1.5 to 1.5, representing z-score standardized species abundance: 0 indicates average abundance, negative values indicate below-average abundance, and positive values indicate above-average abundance.


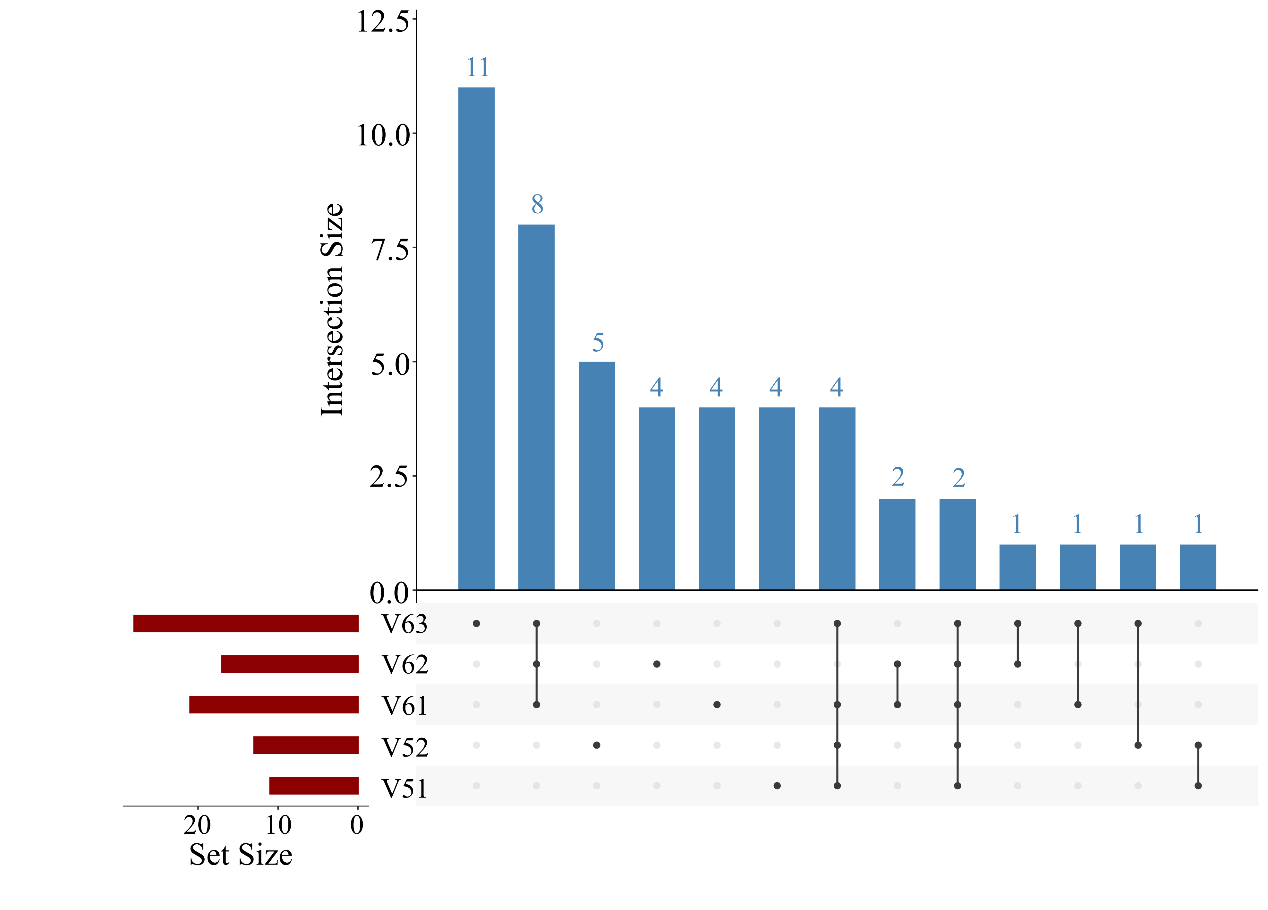


**Fig. S6.** UpSet plot of invertebrates species in different ballast tanks. The red bars represent the number of species in each tank, while the black dots in the matrix indicate individual samples. Connected black dots represent shared species, and the blue bars show the corresponding number of species.


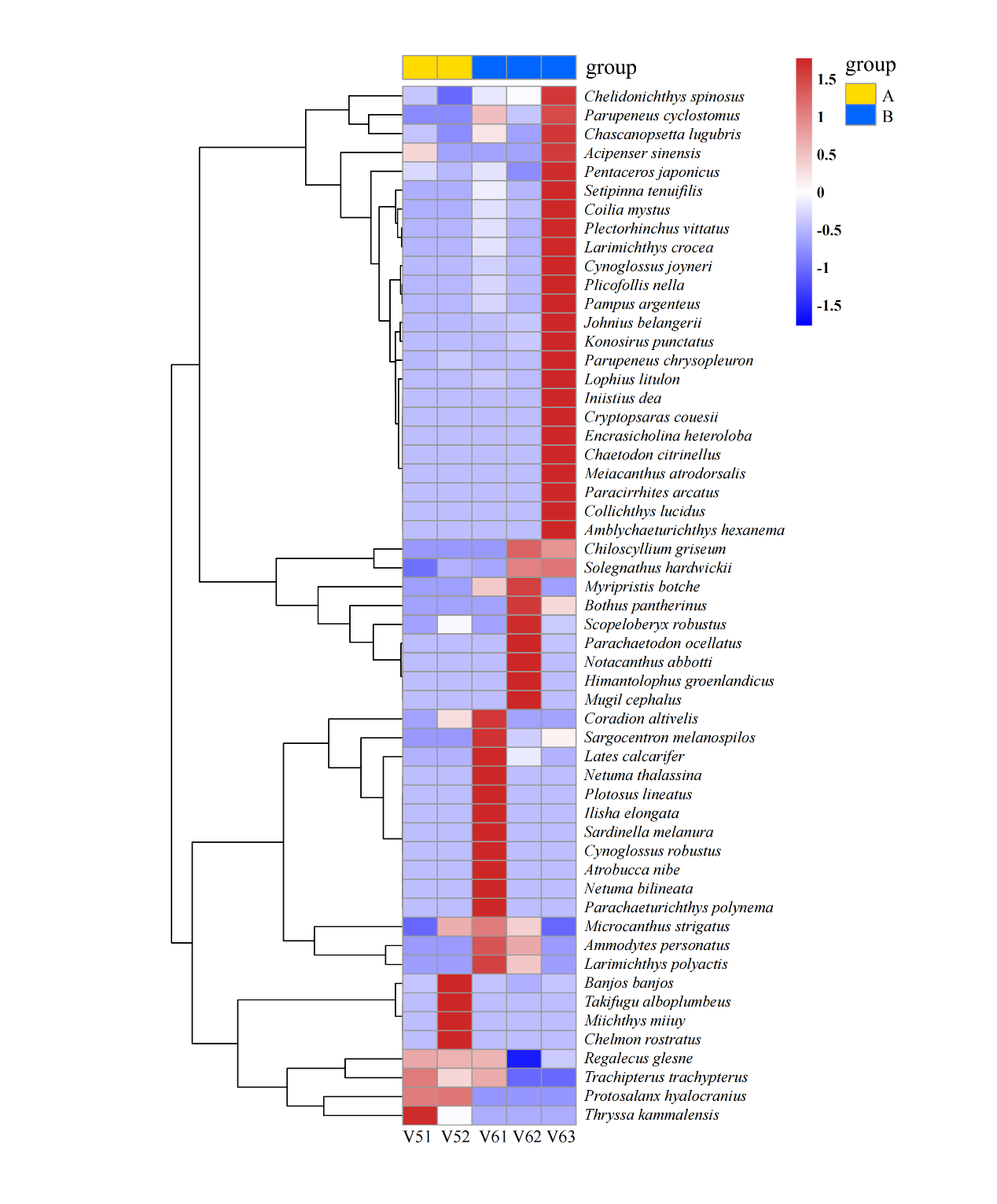


**Fig. S7.** The heatmap illustrates the differences in fish assemblage structure across different ballast tanks. Samples are grouped according to the origin of the ships. Values range from -1.5 to 1.5, representing z-score standardized species abundance: 0 indicates average abundance, negative values indicate below-average abundance, and positive values indicate above-average abundance.


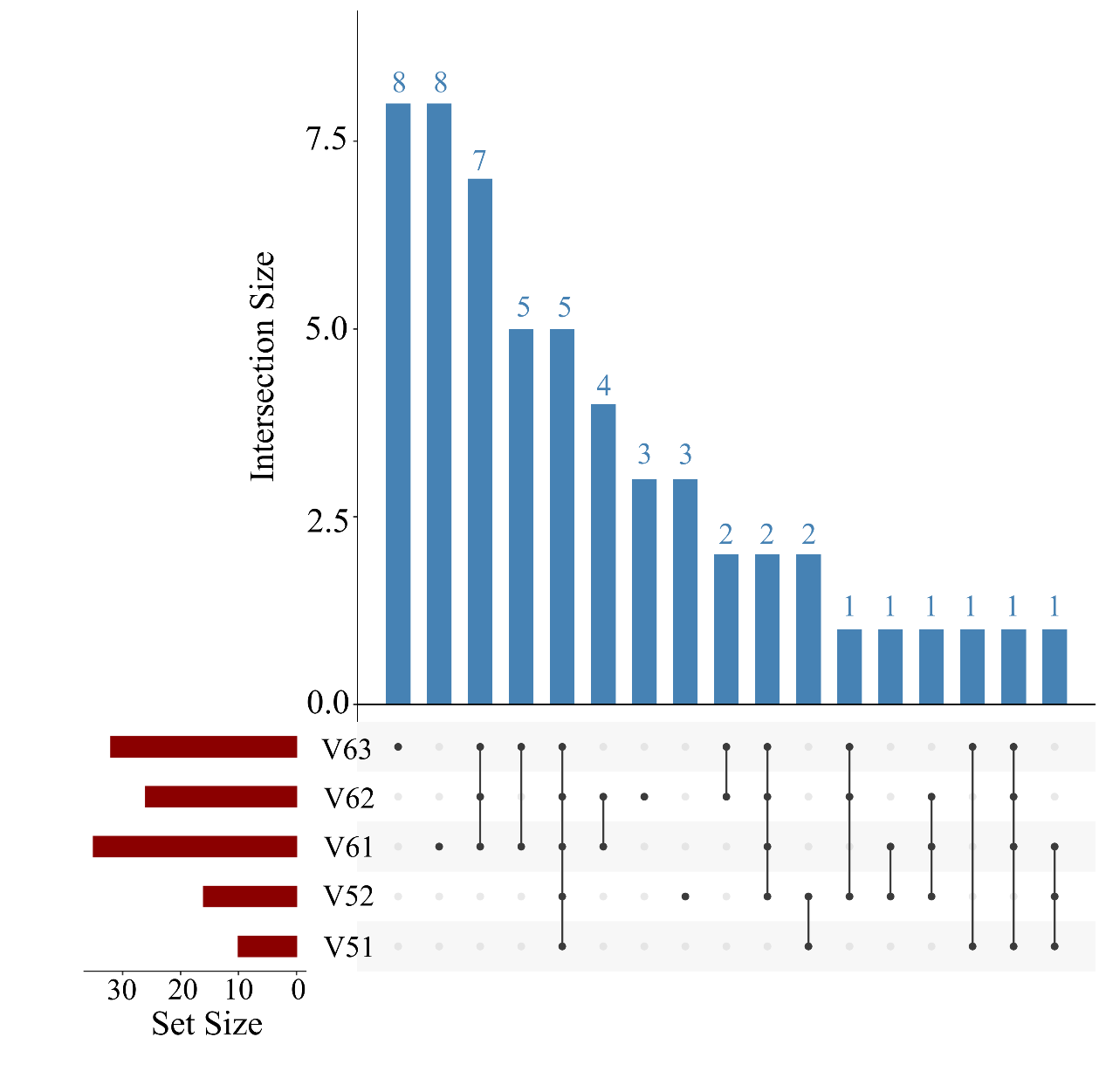


**Fig. S8.** UpSet plot of fish species in different ballast tanks. The red bars represent the number of species in each tank, while the black dots in the matrix indicate individual samples. Connected black dots represent shared species, and the blue bars show the corresponding number of species.

**Table S1.** Sequencing statistics for 18S V4, 18S V9, and 12S.

| 18S V4 |  |  |  |  |  |  |
| --- | --- | --- | --- | --- | --- | --- |
| SampleID | Input | Filtered | Denoised | Merged | Non-chimeric | Non-singleton |
| V1 | 144416 | 129768 | 129176 | 126511 | 119050 | 119050 |
| V2 | 147759 | 136964 | 136385 | 132209 | 130574 | 130572 |
| V3 | 137754 | 120506 | 120384 | 119171 | 118568 | 118568 |
| V4 | 85649 | 78140 | 77954 | 77215 | 76930 | 76930 |
| V51 | 96333 | 87816 | 87700 | 86537 | 86081 | 86079 |
| V52 | 97215 | 88331 | 88061 | 86508 | 85680 | 85678 |
| V61 | 103863 | 97825 | 97255 | 90136 | 87462 | 87459 |
| V62 | 118001 | 110966 | 110228 | 103037 | 100442 | 100440 |
| V63 | 95227 | 89912 | 89484 | 86057 | 84617 | 84615 |
| NC | 146416 | 135921 | 135368 | 131891 | 131293 | 131293 |
| Total | 1E+06 | 1076149 | 1071995 | 1039272 | 1020697 | 1020684 |
| 18S V9 |  |  |  |  |  |  |
| SampleID | Input | Filtered | Denoised | Merged | Non-chimeric | Non-singleton |
| V1 | 91015 | 86075 | 85540 | 83889 | 83826 | 83826 |
| V2 | 77922 | 75050 | 74422 | 72416 | 72413 | 72413 |
| V3 | 146185 | 139998 | 139606 | 136968 | 136709 | 136709 |
| V4 | 136383 | 130432 | 129999 | 126410 | 125978 | 125978 |
| V51 | 143936 | 138311 | 137768 | 131236 | 129768 | 129768 |
| V52 | 106777 | 103645 | 102938 | 101553 | 100570 | 100570 |
| V61 | 139581 | 134468 | 134001 | 130951 | 129491 | 129491 |
| V62 | 127485 | 123030 | 122577 | 119994 | 118299 | 118299 |
| V63 | 142109 | 136573 | 135976 | 128997 | 127729 | 127729 |
| NC | 103937 | 98699 | 98225 | 95845 | 94485 | 94485 |
| Total | 1E+06 | 1166281 | 1161052 | 1128259 | 1119268 | 1119268 |
| 12S |  |  |  |  |  |  |
| SampleID | Input | Merged | Filtered | Non-chimeric | Non-singleton |  |
| V1 | 110293 | 64472 | 62879 | 62260 | 62037 |  |
| V2 | 106408 | 79537 | 76714 | 76613 | 76595 |  |
| V3 | 126025 | 86646 | 84273 | 82977 | 82926 |  |
| V4 | 139438 | 125714 | 121355 | 120948 | 120873 |  |
| V51 | 119009 | 106995 | 102905 | 102537 | 102468 |  |
| V52 | 131989 | 124091 | 119326 | 118852 | 118788 |  |
| V61 | 59609 | 52737 | 51694 | 51366 | 51342 |  |
| V62 | 76856 | 73624 | 72385 | 72194 | 72179 |  |
| V63 | 63336 | 54056 | 53006 | 52580 | 52548 |  |
| NC | 111090 | 44893 | 43060 | 42928 | 42912 |  |
| Total | 1044053 | 812765 | 787597 | 783255 | 782668 |  |
| Note: V1, V2, V3, and V4 represent four ships sampled in chronological order. Three samples from each ship, collected from the same ballast tank, were combined after filtration to form a single sample, each representing one ship. | | | | | | |
| V51 and V52 represent the merged results of three samples from the fifth ship. V51 indicates that two samples were collected from the same ballast tank and combined, while V52 represents the sequencing results of a single sample from a different ballast tank. | | | | | | |
| V61, V62, and V63 represent sequencing results from three samples of the sixth ship, with each sample taken from a different ballast tank. | | | | | | |
| NC denotes the negative control sample, which consists of three ultrapure water samples that were filtered and then combined into a single sample for sequencing. | | | | | | |

**Table S2.** The species of phytoplankton, invertebrate, and fish.

| phytoplankton | invertebrate | fish |
| --- | --- | --- |
| *Achnanthes* sp. BR22.53 | *Paramecium caudatum* | *Anguilla anguilla* |
| *Asterionellopsis glacialis* | *Blackfordia virginica* | *Assessor randalli* |
| *Bacillaria paxillifer* | *Desmophyllum pertusum* | *Thryssa dussumieri* |
| *Bathycoccus prasinos* | *Paramoeba eilhardi* | *Sargocentron violaceum* |
| *Bellerochea* sp. mbrc1 | *Dipsastraea favus* | *Aspidontus taeniatus* |
| *Capsosiphon fulvescens* | *Axinella aruensis* | *Chiloscyllium griseum* |
| *Chaetoceros* sp. RCC4816 | *Pinctada nigra* | *Meiacanthus atrodorsalis* |
| *Chaetoceros* sp. 19 LG-2014 | *Zoanthus gigantus* | *Sargocentron melanospilos* |
| *Chaetoceros affinis* | *Xenostrobus securis* | *Parupeneus cyclostomus* |
| *Chaetoceros* sp. Va7D2 | *Pododesmus macrochisma* | *Paracirrhites arcatus* |
| *Chaetoceros decipiens* | *Ophiura sarsii* | *Etelis carbunculus* |
| *Chaetoceros pseudocurvisetus* | *Styela clava* | *Lates calcarifer* |
| *Chaetoceros didymus* | *Mesodinium rubrum* | *Leiognathus berbis* |
| *Chlorella* sp. ESS-20962 | *Chthamalus challengeri* | *Mene maculata* |
| *Chlorella* sp. BUM11009 | *Calocalanus minutus* | *Halichoeres nigrescens* |
| *Chlorella* sp. ind-29 | *Lafoea dumosa* | *Sardinella melanura* |
| *Chloroparvula pacifica* | *Pontogeneia rostrata* | *Thryssa setirostris* |
| *Chloropicon maureeniae* | *Pseudodiaptomus marinus* | *Arius maculatus* |
| *Chlorosarcinopsis* sp. XLD-T-3 | *Oithona similis* | *Plectorhinchus vittatus* |
| *Cyclotella* sp. | *Acartia omorii* | *Plicofollis nella* |
| *Cyclotella* cf. comensis | *Zoanthus kuroshio* | *Chaetodon citrinellus* |
| *Cylindrotheca closterium* | *Calanus helgolandicus* | *Encrasicholina heteroloba* |
| *Dasya* sp. YY | *Flaccisagitta enflata* | *Talismania antillarum* |
| *Dolichomastix tenuilepis* | *Azumapecten farreri* | *Notacanthus abbotti* |
| *Eucampia zodiacus* | *Muggiaea atlantica* | *Netuma bilineata* |
| *Fragilaria* sp. DM-MEX012 | *Aurelia aurita* | *Aldrovandia affinis* |
| *Grammonema* sp. NE-2015 | *Ditrichocorycaeus affinis* | *Himantolophus groenlandicus* |
| *Guinardia* sp. RCC4799 | *Microsetella norvegica* | *Acanthopagrus latus* |
| *Guinardia striata* | *Temora stylifera* | *Bothus pantherinus* |
| *Helicotheca tamesis* | *Nausithoe punctata* | *Protosalanx hyalocranius* |
| *Leptocylindrus* sp. RCC4827 | *Syllis variegata* | *Clupea pallasii* |
| *Lithodesmium variabile* | *Siphonaria japonica* | *Callionymus lunatus* |
| *Mantoniella* sp. MNURFJ08 | *Oithona simplex* | *Ammodytes personatus* |
| *Melosira moniliformis* | *Oithona plumifera* | *Sebastes pachycephalus* |
| *Micromonas pusilla* | *Euterpina acutifrons* | *Chaeturichthys stigmatias* |
| *Minutocellus* sp. RCC967 | *Achelia assimilis* | *Thryssa kammalensis* |
| *Minutocellus* sp. RCC1954 | *Thaumatomastix* sp. 1 JMS-2012 | *Coilia nasus* |
| *Minutocellus* sp. RCC4809 | *Gruberia* sp. SUAS-2014 | *Sardinella zunasi* |
| *Minutocellus* sp. RCC4472 | *Brachionus plicatilis* complex sp. MNTSA012 | *Plecoglossus altivelis* |
| *Monoraphidium* sp. LUCC 004 | *Adeonella* sp. XJ-2008 | *Seriola dumerili* |
| *Monostroma nitidum* | *Aphroceras* sp. WAM PS0399 | *Nibea albiflora* |
| *Nannochloris* sp. CKM | *Branchiomma* sp. G MC-2013 | *Setipinna tenuifilis* |
| *Navicula* sp. D08_051 | *Callopora* sp. XJ-2008 | *Larimichthys polyactis* |
| *Navicula* sp. RCC4611 | *Chrysaora* sp. JX-2016 | *Chelidonichthys spinosus* |
| *Nephroselmis pyriformis* | *Condylostoma* sp. SUAS-2014 | *Cynoglossus joyneri* |
| *Nitzschia palea* | *Cupressopathes* sp. 2 GB-2006 | *Oplegnathus fasciatus* |
| *Ostreococcus* sp. lucimarinus | *Distichopora* sp. | *Cynoglossus robustus* |
| *Ostreococcus* sp. RCC1120 | *Dysidea* sp. QMG318930 | *Miichthys miiuy* |
| *Phaeodactylum tricornutum* | *Epizoanthus* aff. Corallizoanthus tsukaharai TDS-2010 | *Sebastiscus marmoratus* |
| *Pinnularia borealis* complex sp. | *Euplotes* sp. MdV3B | *Coilia mystus* |
| *Plagiogrammopsis* sp. TN-2014 | *Gastrostyla* sp. Y2 | *Collichthys lucidus* |
| *Pleurosigma strigosum* | *Geodia* sp. JAM-2013 | *Engraulis japonicus* |
| *Psammodictyon panduriforme* | *Grantessa* sp. GW 974 | *Ilisha elongata* |
| *Pseudo-nitzschia multiseries* | *Heliospora* cf. longissima | *Pampus argenteus* |
| *Pseudo-nitzschia americana* | *Isozoanthus* sp. n. TDS-2008 | *Parachaeturichthys polynema* |
| *Pyramimonas* sp. RCC2296 | *Laubierpholoe* sp. A BCG-2017 | *Cynoglossus abbreviatus* |
| *Pyramimonas propulsa* | *Lytocarpia* sp. 1 BP-2015 | *Mugil cephalus* |
| *Pyramimonas* sp. RCC2503 | *Neoparamoeba* sp. COHH 38 | *Lateolabrax japonicus* |
| *Pyramimonas* sp. RCC4805 | *Oithona* sp. Roadstead of Brest | *Regalecus glesne* |
| *Rhodella violacea* | *Onychocella* sp. XJ-2008 | *Cryptopsaras couesii* |
| *Sellaphora* sp. DM-MEX036 | *Palythoa* sp. sakurajimensis MM-2017 | *Chascanopsetta lugubris* |
| *Skeletonema tropicum* | *Paracalanus* sp. Roadstead of Brest | *Chelmon rostratus* |
| *Skeletonema marinoi* | *Pistocythereis* sp. QY-2003 | *Tetrapturus angustirostris* |
| *Skeletonema* sp. RCC1866 | *Placospongia* sp. UCMPWC902 | *Parachaetodon ocellatus* |
| *Spyridia filamentosa* | *Porphyrocrinus* cf. verrucosus POR138 | *Chanos chanos* |
| *Stephanopyxis turris* | *Pseudoparamoeba* sp. | *Coradion altivelis* |
| *Stichococcus bacillaris* | *Tetmemena* sp. Y1 | *Parupeneus chrysopleuron* |
| *Stylonema* sp. RCC2964 | *Tiarina* sp. | *Hexanchus nakamurai* |
| *Thalassiosira* sp. | *Tomopteris* sp. TOMOP_M01 | *Iniistius dea* |
| *Thalassiosira curviseriata* | *Vorticella* sp. 4 PS-2013 | *Terapon theraps* |
| *Thalassiosira tenera* |  | *Benthosema pterotum* |
| *Thalassiosira rotula* |  | *Plotosus lineatus* |
| *Thalassiosira* cf. hispida IAL-2014a |  | *Banjos banjos* |
| *Trebouxia* sp. 142 |  | *Polymixia berndti* |
| *Trentepohlia* sp. TreFl59 |  | *Pentaceros japonicus* |
| *Trieres chinensis* |  | *Planiliza haematocheilus* |
|  |  | *Microcanthus strigatus* |
|  |  | *Trachipterus trachypterus* |
|  |  | *Zeus faber* |
|  |  | *Larimichthys crocea* |
|  |  | *Sirembo imberbis* |
|  |  | *Solegnathus hardwickii* |
|  |  | *Sardinops sagax* |
|  |  | *Conger myriaster* |
|  |  | *Argyrosomus japonicus* |
|  |  | *Atrobucca nibe* |
|  |  | *Netuma thalassina* |
|  |  | *Alectis ciliaris* |
|  |  | *Scopeloberyx robustus* |
|  |  | *Neosebastes entaxis* |
|  |  | *Myripristis botche* |
|  |  | *Amblychaeturichthys hexanema* |
|  |  | *Squatina japonica* |
|  |  | *Lophius litulon* |
|  |  | *Takifugu alboplumbeus* |
|  |  | *Johnius belangerii* |
|  |  | *Konosirus punctatus* |
|  |  | *Scomberomorus niphonius* |
|  |  | *Evynnis cardinalis* |
|  |  | *Acipenser sinensis* |

**Table S3.** Effects of ballast water age and ballast location on species composition of phytoplankton, invertebrate, and fish.

| Factors | Target taxa | F.model | R2 | Pr(>F) |
| --- | --- | --- | --- | --- |
| Ballast water age | phytoplankton | 0.68075 | 0.14544 | 0.9625 |
|  | invertebrates | 0.71833 | 0.15224 | 0.7889 |
|  | fish | 0.40287 | 0.0915 | 0.8792 |
| Ballast locatioon | phytoplankton | 0.96028 | 0.59023 | 0.65 |
|  | invertebrates | 1.1845 | 0.63986 | 0.3 |
|  | fish | 0.88962 | 0.57163 | 0.45 |

**Table S4.** Alien species composition in ballast water samples from different ships (invasive species in bold).

|  | phytoplankton | invertebrata | fish |
| --- | --- | --- | --- |
| V1 | *Thalassiosira curviseriata* | *Zoanthus gigantus* | *Planiliza haematocheilus* |
|  | *Spyridia filamentosa* | *Dipsastraea favus* | *Himantolophus groenlandicus* |
|  | *Skeletonema marinoi* | *Desmophyllum pertusum* |  |
|  | *Micromonas pusilla* | *Axinella aruensis* |  |
|  | *Bathycoccus prasinos* |  |  |
| V2 | *Thalassiosira curviseriata* | *Zoanthus kuroshio* | *Planiliza haematocheilus* |
|  | *Spyridia filamentosa* | *Zoanthus gigantus* | ***Anguilla anguilla*** |
|  | *Skeletonema marinoi* | ***Styela clava*** |  |
|  | *Rhodella violacea* | *Mesodinium rubrum* |  |
|  | *Pyramimonas propulsa* | *Calocalanus minutus* |  |
|  | *Pseudo-nitzschia americana* | *Calanus helgolandicus* |  |
|  | *Nephroselmis pyriformis* |  |  |
|  | *Micromonas pusilla* |  |  |
|  | *Dolichomastix tenuilepis* |  |  |
|  | *Chloroparvula pacifica* |  |  |
|  | *Asterionellopsis glacialis* |  |  |
| V3 | *Thalassiosira curviseriata* | *Zoanthus gigantus* | *Talismania antillarum* |
|  | *Spyridia filamentosa* | ***Styela clava*** | *Regalecus glesne* |
|  | *Skeletonema marinoi* | *Pododesmus macrochisma* | *Netuma thalassina* |
|  | *Pyramimonas propulsa* | *Mesodinium rubrum* | *Netuma bilineata* |
|  | *Micromonas pusilla* | *Calanus helgolandicus* | ***Lates calcarifer*** |
|  | *Dolichomastix tenuilepis* | *Axinella aruensis* | *Encrasicholina heteroloba* |
|  | *Asterionellopsis glacialis* |  |  |
| V4 | *Micromonas pusilla* | *Zoanthus kuroshio* | *Trachipterus trachypterus* |
|  |  | *Paramecium caudatum* | *Talismania antillarum* |
|  |  |  | *Solegnathus hardwickii* |
|  |  |  | *Sargocentron violaceum* |
|  |  |  | *Regalecus glesne* |
| V5 | *Thalassiosira curviseriata* | *Zoanthus kuroshio* | *Trachipterus trachypterus* |
|  | *Spyridia filamentosa* | *Pododesmus macrochisma* | *Solegnathus hardwickii* |
|  | *Skeletonema marinoi* | *Mesodinium rubrum* | *Regalecus glesne* |
|  | *Rhodella violacea* |  |  |
|  | *Psammodictyon panduriforme* |  |  |
|  | *Nephroselmis pyriformis* |  |  |
|  | *Micromonas pusilla* |  |  |
|  | *Helicotheca tamesis* |  |  |
|  | *Asterionellopsis glacialis* |  |  |
| V6 | *Trieres chinensis* | *Xenostrobus securis* | *Trachipterus trachypterus* |
|  | *Thalassiosira curviseriata* | *Paramoeba eilhardi* | *Solegnathus hardwickii* |
|  | *Spyridia filamentosa* | *Mesodinium rubrum* | *Regalecus glesne* |
|  | *Skeletonema marinoi* | *Dipsastraea favus* | *Netuma thalassina* |
|  | *Pyramimonas propulsa* | *Desmophyllum pertusum* | *Netuma bilineata* |
|  | *Pseudo-nitzschia americana* |  | ***Lates calcarifer*** |
|  | *Micromonas pusilla* |  | *Himantolophus groenlandicus* |
|  | *Dolichomastix tenuilepis* |  | *Encrasicholina heteroloba* |
|  | *Chloropicon maureeniae* |  |  |
|  | *Asterionellopsis glacialis* |  |  |
